# EcoBOT: an AI/ML enabled automated phenotyping capability for model plants

**DOI:** 10.3389/fpls.2025.1633557

**Published:** 2025-12-02

**Authors:** Peter F. Andeer, Petrus H. Zwart, Daniela Ushizima, Marcus M. Noack, Lloyd T. Cornmesser, Thomas M. Vess, Zineb Sordo, Stephen Tan, Joseph Zorzi, Chelsea Hernandez, Vlastimil Novak, Yezhang Ding, John P. Vogel, Benjamin P. Bowen, James A. Sethian, Trent R. Northen

**Affiliations:** 1 Environmental Genomics and Systems Biology Division, Lawrence Berkeley National Laboratory, Berkeley, CA, United States; 2 Center for Advanced Mathematics for Energy Research Applications, Lawrence Berkeley National Laboratory, Berkeley, CA, United States; 3 Berkeley Synchrotron Infrared Structural Biology Program, Lawrence Berkeley National Laboratory, Berkeley, CA, United States; 4 Molecular Biophysics and Integrated Bioimaging Division, Lawrence Berkeley National Laboratory, Berkeley, CA, United States; 5 Applied Mathematics and Computational Research Division, Lawrence Berkeley National Laboratory, Berkeley, CA, United States; 6 Berkeley Institute for Data Science, University of California, Berkeley, Berkeley, CA, United States; 7 Bakar Institute, University of California, San Francisco, San Francisco, CA, United States; 8 Joint Genome Institute, Lawrence Berkeley National Laboratory, Berkeley, CA, United States; 9 Joint Bioenergy Institute, Lawrence Berkeley National Laboratory, Berkeley, CA, United States; 10 Department of Mathematics, University of California, Berkeley, Berkeley, CA, United States

**Keywords:** automated, self driving, Gaussian Process, plant phenomics, AI image analysis

## Abstract

**Introduction:**

Advances in automation and AI/ML offer new opportunities for plant science, including design, modeling, and analysis. This study aimed to develop an automated platform for researching small model plants under axenic conditions and integrate it with AI/ML tools.

**Methods:**

The EcoBOT platform was developed, which consists of sterile containers (EcoFABs) for growing plants and imaging for monitoring plant growth and health. *Brachypodium distachyon* was grown on the EcoBOT, and its response to nutrient limitation and copper stress was evaluated.

**Results:**

The results showed that Brachypodium distachyon grown in the EcoBOT maintained sterility and responded to nutrient limitation and copper stress. Analysis of over 6,500 root and shoot images revealed varying sensitivity and response rates to copper. Bayesian Optimization was used to improve model accuracies relating copper concentrations to plant biomass via sequential experiments, resulting in a >30% improvement.

**Discussion:**

The findings of this study demonstrate the potential of the EcoBOT platform for researching plant responses to environmental factors. Future experiments could focus on relating other chemical stresses and microbial interactions to create generalized models of plant responses.

## Introduction

Model plants are critical for foundational scientific research and have been used to establish many of the known links between genetics, environmental variables and plant phenotypes. These plants are typically selected based on a number of factors including their genome sizes, physical size and growth rate (Arabidopsis Genome Initiative, 2000; Koornneef and Meinke, 2010; Brkljacic et al., 2011). However, in order to effectively establish causality between treatments and phenotypes, it is often important to maintain sterile conditions given the major impact microbes can have on plant phenotypes (Kuijken et al., 2015; Müller et al., 2016). Recently, we reported using EcoFAB 2.0 devices as a sterile growth system for characterizing plant phenotypes under sterile conditions suitable for gnotobiotic research (Novak et al., 2024a).

However, even with model plants, research can be slow and complicated because the links between plant phenotypes and factors such as their genetics and environmental conditions may be obscured by uncontrolled experimental variability (Poorter et al., 2012). These challenges are exacerbated when data are collected using different methods. In addition, while model plants are often small and fast growing, it can take several weeks to go through the necessary stages of their development for the appropriate observations to be made (Watt et al., 2009; Brkljacic et al., 2011).

Automated growth and plant phenotyping systems have the potential to accelerate plant research by making experiments more efficient for example through Bayesian Optimization of data models (Shahriari et al., 2016; Rincent et al., 2017; Tanaka and Iwata, 2018; Greenhill et al., 2020). Similar strategies have been used in a number of other research areas with autonomous laboratories engaged in material science (Szymanski et al., 2023) and protein research (Rapp et al., 2024), and to make time-sensitive experiments (e.g., beamline analyses using advanced light sources) more productive (Noack et al., 2019; Melton et al., 2020; Noack et al., 2020). While AI/ML tools, including Bayesian Optimization strategies, have been effectively employed to guide plant breeding strategies (Crossa et al., 2025), relatively few studies (Nikitin et al., 2019) (Khan et al., 2024) have leveraged these approaches for automated experimental design, data analysis, and modeling the impacts of environmental stressors on plants.

There is also a lack of compact systems for phenotyping model plants under sterile conditions. Phenotyping facilities developed for agricultural crops (e.g., Advanced Plant Phenotyping Laboratory (APPL, www.ornl.gov/appl), GrowScreen-Rhizo (Nagel et al., 2012), HT3P (Li et al., 2020), Field Scanalyzer (Virlet et al., 2016), Bellwether Phenotyping Platform (Fahlgren et al., 2015) and PhenoLab (Amby et al., 2025) are extremely powerful facilities that will likely lead to a number of important observations in plant research. While there are exceptions (Wu et al., 2018; Hüther et al., 2020), these are often 100s of meters in size up to entire field sites, and use complex, expensive machinery to move plants; they are not designed or necessarily suitable for studying the small model plants used by many researchers. Importantly, these systems are not designed to exclude foreign microorganisms. While direct translation between model plants and bioenergy crops is challenging, much of our understanding comes from model plants like *Brachypodium distachyon*, and their importance to fundamental research is unlikely to diminish. Sterile systems suitable for model plants such as the *Brachypodium* are therefore needed.

To take full advantage of AI/ML experimental design it is important to capture plant phenotypic data in a scalable, reproducible manner. While there are a wide-array of modalities used in automated plant phenotyping, imaging is by far the most common. Integrating automated image acquisition with the analyses and plant care during growth has the ability to greatly expedite the evaluation and discovery of the underlying causes of specific traits. Improved imaging and analysis has helped to automate plant phenotyping since these methods are relatively fast and non-destructive. For plant shoot growth, hyperspectral imaging has become popular for monitoring everything from single-plant images taken in pots to satellite images of entire fields (Ge et al., 2016; Gao et al., 2017; Perez-Sanz et al., 2017; Maes and Steppe, 2019; Tayade et al., 2022). Analyses of hyperspectral data continue to expand beyond plant growth to include the identification of plants stressed through nutrient deficits and infections (Zhao et al., 2005; Maes and Steppe, 2019). For plant roots, images can be acquired from field plants through *in situ* rhizotrons (Bates, 1937) and excavated plants (York et al., 2022). In the laboratory, plant roots can be visualized using various non-destructive methods that range from seedlings grown on plates (Costa et al., 2000), microfluidic devices (Grossmann et al., 2011), and dedicated imaging systems for specialized analyses (Jahnke et al., 2009; Clark et al., 2011; Rellán-Álvarez et al., 2015; Shao et al., 2021). Incorporating image processing into automation is thus an essential step to automate plant phenotyping. A wide variety of software packages have been developed to quickly process these images (Teramoto and Uga, 2022) including PlantCV (Gehan et al., 2017) and RhizoVision (Seethepalli et al., 2021). Two such programs are RhizoNet (Sordo et al., 2024) and EcoSpec (Zwart et al., 2025), which use AI/ML pipelines specifically designed to automate the analysis of EcoFAB 2.0 root and shoot images, respectively.

Beyond their engineering and analytical capabilities, automated phenotyping platforms open new opportunities to study the mechanisms that underlie plant development and adaptation (Rahaman et al., 2015). Continuous, non−destructive measurements have the potential to capture transient plant trait changes that reflect shifts in resource allocation, growth strategy, and stress response (Al-Tamimi et al., 2022). When performed under defined or axenic conditions, observations reveal baseline patterns of growth and development without the confounding effects of microbial communities and controlled addition of stressors such as nutrient deprivation or heavy metals can help isolate specific physiological and morphological responses, particularly when linked to plant genomics (Pratap et al., 2019). Ideally, when below ground data can be collected and integrated over time, this context can help provide the context needed to link trait variation to ecological function, improving our understanding of how plants cope with environmental change (Yang et al., 2020).

The need for efficient and automated plant phenotyping systems has driven the development of the EcoBOT, an automated platform for the growth and analysis of model plants within EcoFAB 2.0 devices under defined conditions. To validate the EcoBOT’s capabilities, we first investigated the growth of the model grass *B. distachyon* under axenic conditions and evaluated its response to nutrient deprivation. We then examined the following aspects of the system 1): use AI/ML tools to design experiments, analyze images, and generate phenotypic models of *B. distachyon* 2); to investigate the effects of copper, which can persist in soils through past agricultural and industrial practices (Rahaman et al., 2015; Al-Tamimi et al., 2022; Zwart et al., 2025), stress on *B. distachyon* biomass and shoot health; and 3) to apply advanced data analysis techniques to extract insights into plant growth and health. Gaussian Processes were used to model *B. distachyon* root and shoot sizes to copper stress, Bayesian Optimization to optimize experimental design, and AI/ML-driven analysis software (RhizoNET and EcoSpec) to extract these metrics from images. Furthermore, we employed a hybrid unsupervised learning approach to analyze time-series data and gain deeper insights into the impacts of copper exposure on specific *B. distachyon* phenotypic traits.

## Materials and methods

### Experimental design

Plant experiments were performed on the EcoBOT, a 2 m x 2 m system consisting of a liquid handling robot, growth chamber that can hold > 150 EcoFAB 2.0 devices, robotic arm and imaging stations for plant phenotyping. The model plant, Brachypodium distachyon, was grown for up to 30 days for these experiments, however plant species and experimental duration can vary and are typically dictated by the EcoFAB 2.0 dimensions which have a 10 ml root zone and 50 mm chamber height.

**Figure 1:**
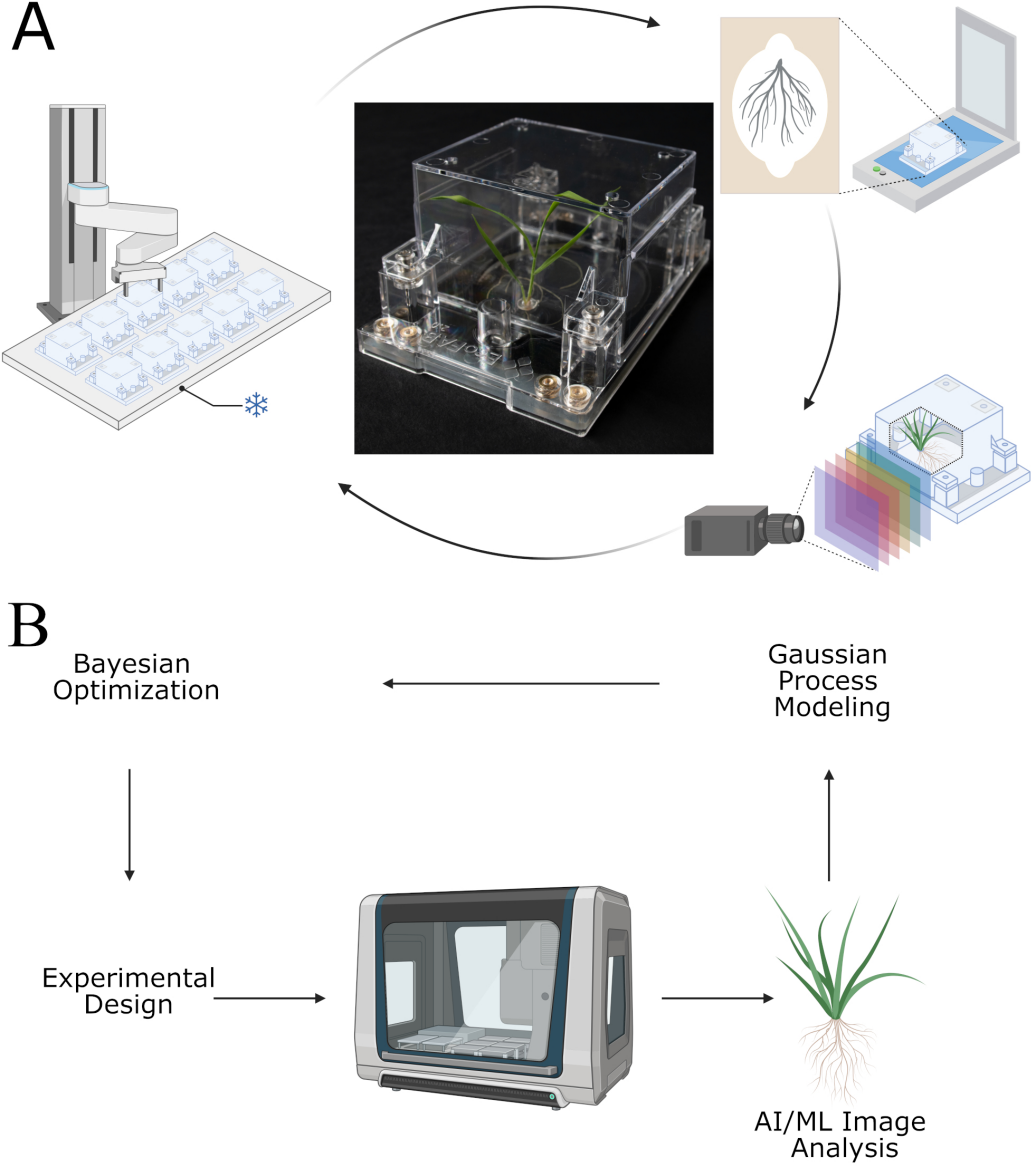
The EcoBOT is an experimental system for small model plants with integrated growth chamber, liquid handling unit and imaging stations. **(A)** For phenotyping, EcoFABs are transported from the growth chamber to a flatbed scanner and hyperspectral camera. **(B)** Overview of how the EcoBOT can be used in AI/ML driven experiments. Experiments designed using Bayesian Optimization are run on the EcoBOT where plant root and shoot images are collected and then analyzed to extract information for Gaussian Process Models. These models are then used to design new experiments through Bayesian Optimization. BioRender was used in part to generate this image.

**Figure 1** displays (A) the basic operations of the EcoBOT as used in this study and (B) the logic for EcoBOT experiments. A video demonstrating the system can be seen at: https://eco-fab.org/. The objectives of this study were 1) to validate that we could grow aseptic model plants on the EcoBOT under stress conditions, 2) to confirm that we could use the EcoBOT to make GP-models of the impacts of stressors on plants, 3) that we could use these models to inform subsequent experiments that would improve these models, 4) verify that we could use image data collected throughout the experiment to track plant growth and health and 5) determine how this time series information could be used to gain further insight into the impacts of stressors on plant growth. **Figure 1** was in part generated using BioRender (Andeer, P. (2025) https://BioRender.com/z67e748 & Andeer, P. (2025) https://BioRender.com/ebp6ylj).

### EcoBOT equipment and control information

The EcoBOT liquid handler is a Hamilton Vantage 2.0 with 8x 2 ml and 2x 5 ml channels, quad core gripper and tilt module for pooling liquid in the EcoFAB 2.0 root chamber for removal. A HEPA hood is mounted above the liquid handler and the unit is housed in a cabinet that includes lower and rear logistics cabinets which have been converted into plant imaging stations and a plant growth chamber, respectively. In between the front of the unit with the liquid handling unit and the rear logistics cabinet is a 1.16 M HMotion extended reach robotic arm (OTP‐PRSA‐0016) mounted on a linear axis rail.

#### Growth chamber

The growth chamber is built into the rear logistics cabinet and consists of 3 custom shelves built from anodized black aluminum (Machining Unlimited; Dublin, CA; **Supplementary Figure S1**) with 57 spaces accessible for automated processing per shelf. The shelves are spaced vertically to allow the arm to lift an EcoFAB 2.0 from the back without disturbing the devices in front. Reflective and black window covering was added to the cabinet doors to minimize outside light from entering the unit.

**Figure 2:**
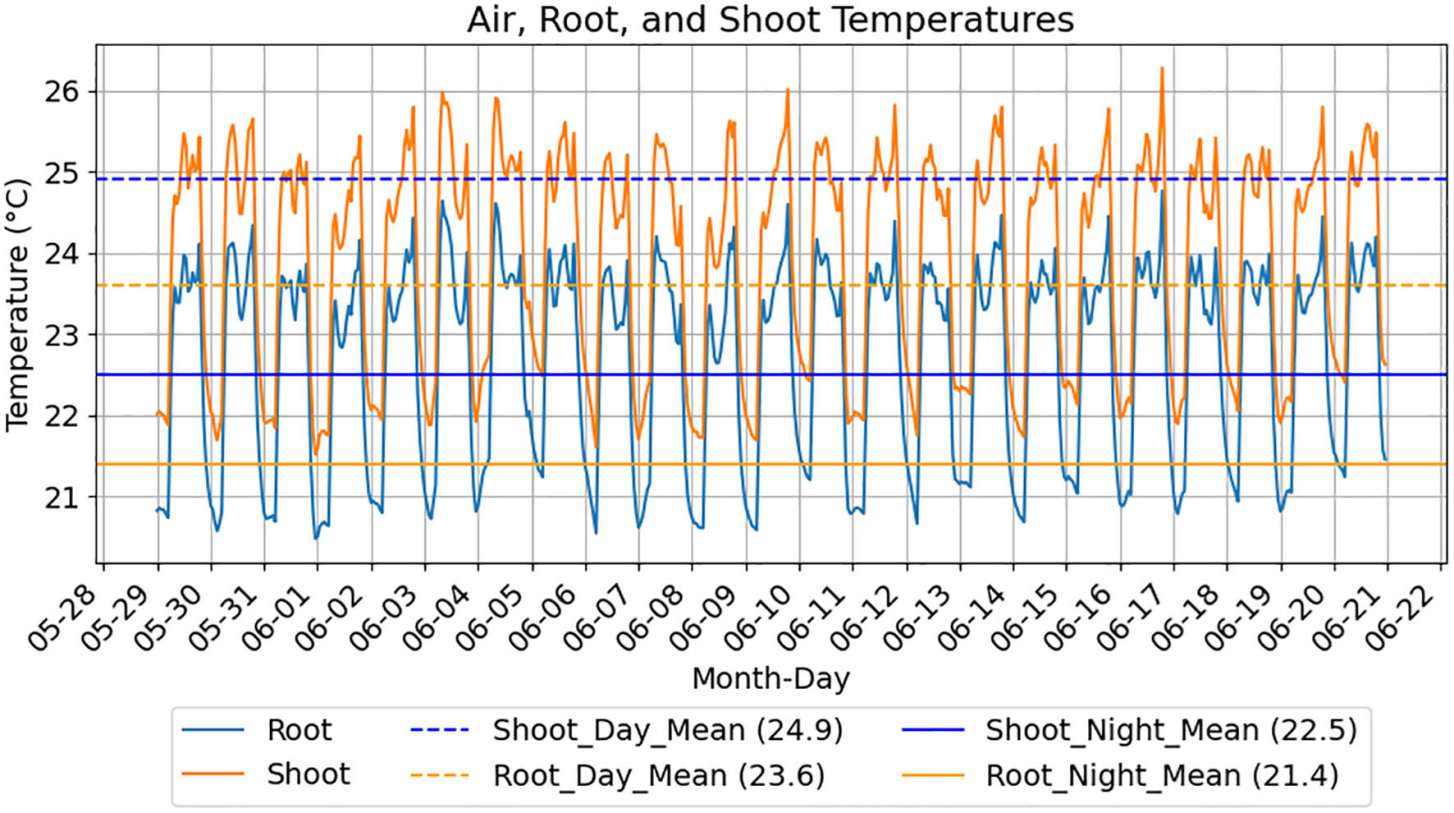
EcoFAB root and shoot temperature estimates over experimental diel cycles. Measurements for an EcoBOT experiment using a 14 hr light cycle are displayed. Root and shoot chamber temperature estimates are produced from 2 EcoFABs positioned on the experimental shelf with RTD probes in the lower and upper EcoFAB chambers, respectively. Root chamber temperatures follow the same patterns as the shoot temperatures but are consistently lower due to contact with the cooled shelf.

Fresh air is supplied to the chamber by the HEPA filter above the liquid handling robot and is circulated by fans mounted on each shelf. Coolant (Dynalene LC-PG 50%; Whitehall, PA) is also circulated through thin (18 gauge) stainless steel heat exchangers (25.5” x 66”; Omega Thermo Products; Stratford, WI) installed between the shelves and its supports by dedicated 7 L refrigerated baths (Polyscience; Niles, IL) set at 17 °C and 60% speed for these experiments. Eight RTD probes (SA1-RTD-80; Omega Engineering; Norwalk, CT) are mounted on each shelf in the following locations: air temperature is monitored by 2 probes are approximately 1” below and between the 1st and 2nd and between the 3rd and 4th LED lights, shelving temperature is monitored by 2 probes taped to opposing corners of the shelf and EcoFAB root and shoot temperatures are estimated by 2 EcoFAB 2.0 devices placed in inaccessible locations in the back row of each shelf with a probe in the root chamber and another in the shoot chamber. MadgeTech 4 software connected to dataloggers (OctRTD 8 and RTD TempX8; MadgeTech; Warner, NH) record temperatures every 5 minutes. **Figure 2** displays average root and shoot temperatures from an EcoBOT experiment. Growth chamber lighting is supplied by 4 PhytoFY RL LED lights (Osram; Premstätten, Austria) mounted on each shelf which are operated using the product’s software (v.1.0.22).

#### Imaging stations

Root scans were performed on a flatbed scanner which are widely used for imaging plant roots (Costa et al., 2000; York et al., 2022). Specifically, root images were captured on an EPSON Perfection V850 Pro Flatbed Photo Scanner using the EPSON scan software at 48-bit color and 600 dpi that were trimmed to a 3.4” x 5” image around the EcoFABs. Hyperspectral images were acquired by a Specim IQ (Specim; Oulu, Finland) camera (400–1000 nm in 205, 7 nm bins) operated using Specim IQ Studio. The camera was mounted onto a custom motorized actuator located in the lower logistics cabinets to capture images from multiple angles which is important for grasses (Poiré et al., 2014). The distance from the camera lens to the center of the EcoFAB 2.0 in the turnable carriage is approximately 9.5". Calibration and focus was set prior to all experimentation and the former refreshed as needed. A 29 ms integration time was used for all images. The EcoFABs were illuminated during the imaging with a pair of Wellmaking HL-300 40W 5600K LED lights with the white and amber filters along with a supplemental light built with blue, cyan and white LED bulbs along with a 50W Halogen bulb. The cabinet door housing the hyperspectral camera is blacked out with vinyl to prevent outside light from interfering with the imaging.

#### Control software

General control of the EcoBOT during experiments was done using the Hamilton Run Control Software (4.4.0.7740) running on Hamilton Venus for Vantage v1.8.3.0. Initial training of the HMotion arm was performed using the system’s adaptive training methods and then refined manually for each shelf position to within 0.1 mm to ensure reliable transport. During experimentation, a program is run to close the gate on the liquid handling unit and initiate the HEPA filter above the liquid handling unit (2,100 RPM) which remains on throughout the experiment. EcoFABs are loaded onto the shelves via the hyperspectral turntable where the HMotion arm transports it to the appropriate location in the growth chamber. An initial, supervised session is used to ensure the EcoFABs are delivered reliably to the EPSON scanner and refinements are made to the shelf positions as needed.

To reduce jostling of the plants, the plants were transported at a relatively slow speed of ~120 mm/s. At this speed, it took approximately 10 minutes per EcoFAB to transport and collect images. Imaging was performed in carriages at each station which streamlined analyses and made comparative and time-series data analyses possible since image alignment was consistent. For example, the average deviation in the alignment of a subset of 40 images used in this study was less than 2 pixels in any direction (Zwart et al., 2025). For each imaging run, an external program within the native Hamilton Run Control software turns on the camera and hyperspectral lights and then a CSV (comma-separated values) file with the list of EcoFABs and corresponding shelf positions is loaded and cycled through until all of the images are collected before shutting down the camera and lighting. Barcodes on each EcoFAB are scanned prior to imaging and then imaging files and folders were automatically named using the EcoFAB barcode, date-time information and the image angle.

The flatbed scanner and hyperspectral camera are operated through custom macros generated using Pulover’s Macro Creator (https://www.macrocreator.com/) which were converted into executable files run by the run control software. Executable programs turn on and off the hyperspectral lights and camera via an Arduino controller (v. 2.1.1) and the camera movement via Snap2Motion 2.0 which operates the linear actuator (ModuSystems; San Jose, CA).

#### *B. Distachyon* experiments

##### EcoFAB 2.0, seed sterilization, media preparation and harvest

EcoFAB 2.0 devices were sterilized and assembled as previously described (Novak et al., 2024a) and detailed in the protocol “Assembly and sterilization of EcoFAB 2.0 for plant growth experiments” (https://www.protocols.io/view/assembly-and-sterilization-of-ecofab-2-0-for-plant-c5gty3wn.html) with a few exceptions: 1) instead of airpore tape and septa, the liquid ports of the EcoFAB 2.0 were covered by custom 3D-printed caps (**Supplementary Figure S2**) which prevent contamination from outside air, but can be lifted by the robot through the use of a suction-cup tool and 2) to reduce occlusion of the plant shoots during hyper-spectral imaging, clear membranes that permit airflow (Breathe-EASY #BMTM-1000; Diversified Biotech) were used to cover the ports on the top of the EcoFAB 2.0 plant chamber. *Brachypodium distachyon* Bd21–3 seedlings were surface sterilized as previously described (Novak et al., 2024a). After 3 days of stratification at 4 °C and 3 days of germination on 1% water agar plates, seedlings were placed into EcoFABs filled with 10 ml of media within a biosafety cabinet and loaded onto the EcoBOT. During growth, transpired media was replenished as needed using sterile, MilliQ or LC-MS grade water. This was done during CuSO_4_ amendment in the 25 day copper stress experiments and twice during the 30 day nutrient experiment.

All experiments used dilute Murashige and Skoog (MS) media (Murashige and Folke, 1962). For the nutrient stress experiment 50% MS salts media was prepared from chemical stocks using LC-MS grade water and composed as outlined in **Supplementary Table S1**. For the copper stress experiments, 30% MS media was made from dehydrated powder (1.3 g/L, Caisson Labs MSP01; Smithfield, UT). In all experiments, pH was adjusted to 5.9 +/- 0.1 with sodium hydroxide and filter sterilized. CuSO_4_ solutions were prepared in 0.5 ml of 30% MS media and unamended media was added to the control plants on the 10th day of growing on the EcoBOT.

At harvest, EcoFABs were visually inspected for biofilm or other signs of microbial growth before removing an aliquot (50–100 ul), mixing it with 100 ul of R2A media (Reasoner and Geldreich, 1985) and incubated at 27°C for at least a week in multiwell plates where they were monitored for growth. *B. distachyon* was then removed from the EcoFABs, the remaining seed parts were removed and shoots were separated from the roots using tweezers, patted dry and weighed on a balance.

##### Lighting settings for the experiments

The spectral distribution of the lighting was selected using the average color ratios of light recorded over 19 clear days in June of 2018 and 2019 at the Eugene, Oregon station run by the University of Oregon’s Solar Radiation Monitoring Laboratory (Peterson and Vignola, 2017). The spectral irradiances were converted to photon flux densities for each color bin over the course of a day and ratios were calculated relative to the red light bin. These averages were relative to Red light: Red/UVA: 13.92, Red/Blue: 1.29, Red/Green: 1.07, Red/Far Red: 1.38.

For the nutrient stress experiment, the 4 PhytoFY RL LED light intensity levels for UVA (380–400 nm), blue (400–500 nm), green (500–600 nm), red (600–700 nm), far red (700–780 nm) and general white were set at 26, 25, 60, 20, 51 and 35, respectively, which provided an estimated average of PFD 350 μmol/m²/s on a 14/10 hr light on/off cycle (**Supplementary Figure S3**). For the copper stress experiment, these were adjusted to 10, 20, 43, 13, 38 and 35 (**Supplementary Figure S4**). While the light intensity estimates for the nutrient stress experiment were generated from standard curves, prior to the copper stress experiment, prism diffusers were installed on each LED light fixture to improve dispersion. So the intensities in **Supplementary Figure S4** for each shelf position were measured manually using these exact settings and an EcoFAB 2.0 chamber was placed above the spectrometer to account for light filtered by the chambers (~9 - 12% depending on wavelength) which accounts for the lower intensities reported in those experiments. All light measurements were made using a LI-COR LI-180 Spectrometer (Lincoln, NE).

##### Image processing

Two imaging software packages on NERSC (National Energy Research Scientific Computing Center) were used to process the root scans and hyperspectral images, respectively: RhizoNET (Sordo et al., 2024) and EcoSPEC (Zwart et al., 2025).

RhizoNet (Sordo et al., 2024), processes color scans of plant roots cultivated in EcoFABs using a supervised AI/ML algorithm that enables researchers to detect root area from digital images with precision. At the core of the system, an advanced Residual U-Net architecture turns digital scans from EcoFAB’s bottom area into root images; this algorithm is an enhancement of the original U-Net model for semantic segmentation. To allow for efficient processing of large, high resolution images, images were partitioned into 64x 64 patches. This improvement introduces residual connections at each level of resolution in both the encoder and decoder pathways, greatly boosting the accuracy of root segmentation tailored for EcoFAB conditions. Additionally, RhizoNet incorporates a convexification procedure to effectively encapsulate roots identified over time series, enabling the precise separation of primary root components from complex backgrounds. For this study RhizoNET on NERSC was trained on 1 NVIDIA Tesla V100 GPU node (compute time between 30 min and 1h30 min). Because the RhizoNET models were initially trained using 88,102 overlapping patches from 5 different EcoFAB 2.0 experiments (Sordo et al., 2024), only a small subset of 19 root scans with particularly complex backgrounds were manually annotated from these specific experiments and added to the model test/train sets to assist to fine-tune the models used in these analyses.

The hyperspectral data were processed as described using EcoSpec (Zwart et al., 2025), an AI/ML tool for analysis of the hyperspectral imaging data to identify spectral features that are altered in response to experimental conditions. These were preprocessed by eliminating spectral ranges below 430 nm and above 800 nm due to low-quality of signal which still allows for NDVI and other plant indices to be calculated. Each spectral image, originally sized at 64×512×512, was reduced to a 3×512×512 image using a spectral projector head. This dimensional reduction was achieved via a sequence of three fully connected layers, each followed by batch normalization and ReLU activation. The reduced representation was then passed through a random sparse mixed-scale convolutional network (Roberts et al., 2024) to yield a two-class probability map. Seven independent networks, including the initial projector heads, were trained end-to-end on ten partially labeled images of plants throughout their growth cycle. For inference, the resulting class probability maps were averaged and renormalized to generate labels. Due to the EcoBOT’s precise alignment of the EcoFAB 2.0 in the hyperspectral imaging stage, combined with a Fourier-based alignment step to a reference mean EcoFAB image, we could apply a static mask that eliminates spurious labels outside a predetermined area of interest, thus making the assignment more robust. The total number of shoot pixels per independent view was determined by counting all non-background pixels within the region of the EcoFAB plant growth chamber where plants can grow.

For each plant, spectral characteristics were extracted from each pixel based on the segmentation method described above. A mean plant-pixel spectrum was computed for each view. These spectra underwent sparse dictionary learning, where each spectrum was decomposed into two components constrained to closely match observed data, facilitating a straightforward interpretation of the decomposition output. In this case, the resulting components clearly distinguish between ‘green’ and ‘non-green’, allowing us to compute the fraction of healthy versus unhealthy pixels per view.

NDVI values, that have previously been shown to correspond to healthy plants based on mapping to RGB images (Zwart et al., 2025), were calculated for each pixel were performed using Equation 1 where *m750* and *m550* represent the mean reflectance between 740 and 750, and 540 and 560, respectively and *mmin* is the minimum reflectance for the pixel. For each image, pixels were then binned (n=50) which were used to calculate the average values for each plant, day (**Supplementary Data**).

(1) $$NDVI\;=\left(\frac{\left(m750\;-\;m550\right)}{\left(m750+m550+2*mmin\right)}\right)\;$$

##### Gaussian process modeling

To compute the Gaussian Process (GP) model, perform inference, and determine model performance, including NRMSE values, the GPOptimizer method was run within gpCAM (https://gpcam.lbl.gov/) with parametric but constant noise function, stationary ARD (automatic relevance determination) kernel, and initial lengthscale bounds set to 1% and 5X the lowest and highest observable values in the datasets. Repeated generations of models using these bounds were then used to help refine those values. Markov chain Monte Carlo optimization was used with a maximum number of iterations between 10,000 and 25,000. To compare model performances, 30 test-train datasets were created by making 5 iterations of 6-fold cross validation, utilizing stratified sampling for each model iteration (**Supplementary Data**). Stratification of the data was performed by binning sample sets into 3 based on copper concentrations using the KBinsDiscretizer function in sklearn.preprocessing module. This process was used to maintain representative distributions of copper concentrations and the data was shuffled between iterations.

NRMSE and CRPS values were calculated using the RMSE and CRPS functions within gpCAM using both the full dataset (‘full’) or paired test-train datasets with the RMSE normalized by dividing by the range of values in the evaluated dataset (i.e., the range of each test set was used to normalize that set of RMSE values). Full_NRMSE_ratios values (**Supplementary Data**) were produced by dividing the NRMSE values of the real dataset by values calculated using scrambled y-values for a given evaluation set and the NRMSE ratios to determine how much better the data fit the actual dataset relative to a randomized one. Posterior means and covariances (with and without noise) were then converted into standard deviations and calculated from 0 to 500 **μ**M CuSO_4_ in steps of 1 **μ**M. 95% confidence intervals (95% CI) for test set NRMSE values were performed using 1000 bootstrapped datasets. Models were generated using either CuSO4 concentrations as the sole input variable or all variables (EcoFAB specific lighting intensity measurements and experimental temperature estimates) provided in the **Supplementary Data**. NRMSE outputs were compared using a Student’s T-tests to compare test-set NRMSE values.

For the Bayesian Optimization, experimental conditions (i.e., copper concentrations) were selected by running the gpcam.ask() function within gpCAM using the ‘variance’ acquisition function based on an initial draft of the root model for the 1st experiment and requesting 5 new concentrations to evaluate.

To evaluate potential batch effects between the two experiments, Student’s T-tests were performed for root and shoot biomass data for the control condition shared between the data where no copper was added.

### Statistics and other data processing

Except where indicated, all post-processing of data was performed in Python (v. 3.11.10 or 3.11.11) and images were generated using the MatPlotLib and/or Seaborn packages. Significant differences for the nutrient experiment were determined by performing an ANOVA across conditions for each of root and shoot mass using using the f_oneway function from scipy.stats (v. 1.13.1) and pairwise comparisons performed with the pairwise_tukeyhsd function from the statsmodels package (v. 0.14.4).

For the pilot copper experiment, Student’s T-tests were performed using the ttest_ind() function within scipy.stats between copper and control plants since there was only comparison for each of roots and shoots.

Daily time series data for roots and shoots were generated by smoothing and interpolating the pixel count outputs from RhizoNET and EcoSpec, respectively for days 4 through 25. Euclidean normalized pixel counts (simplified to shoot pixels) were calculated by squaring the counts from each of the three images for each plant, time point (top, side and front), summing them and taking the square root. Health fraction datasets were generated by calculating the average of the health fraction data from each angle weighted by the respective pixel counts. NDVI values were calculated by calculating the average value from the binned values across all imaging angles. These data were all then smoothed using Savitzky-Golay filter (Savitzky and Golay, 1964) where the savgol_filter() function from scipy.signal was used with window lengths of 7 and polyorder 5 for shoot health and NDVI and polyorder 3 for root and shoot pixels, selected based on visual inspection of fits and then interpolated using the BSpline() function from scipy.interpolate.

Pearson correlation coefficient and R^2^ values between raw and scaled pixel data and fresh weights was performed using the pearsonr() function from scipy.stats with confidence intervals calculated on the Fischer’s z-transformation of the data. The Pearson correlation coefficients of the time series data was calculated using the corr() function in pandas and confidence intervals determined by the 2.5th and 97.5th percentile values of 10,000 bootstrapped datasets.

Significant differences between dosings vs non-dosed controls was determined for each day by performing an ANOVA and Tukey *post-hoc* comparison with multiple test correction using the pairwise_tukeyhsd() and multipletests() functions from the statsmodels package (v. 0.14.4) and then filtering out comparisons that did not involve the control condition (0 **μ**M CuSO_4_).

For UMAP dimensional reduction, projection and clustering the scaled (MinMax scaling across experimental data sets) root and shoot pixels and health fraction data for days 11 through 25 (the period of time when plants were exposed to CuSO_4_) were combined and reduced using the UMAP function from umap-learn (v. 0.5.7) with the following settings (n_neighbors=5, min_dist=0.05). HDBSCAN clustering was performed using the function of the same name from the hdbscan package (0.8.40) using min_cluster_size of 8 and min_samples of 3. ANOVA of the various parameters was performed using the f_oneway function from scipy.stats and pairwise comparisons performed with the pairwise_tukeyhsd function from the statsmodels package (v. 0.14.4).

## Results

Given the small size of model plants and to maintain a small footprint suitable for a typical laboratory, the EcoBOT was designed to incorporate a growth chamber and imaging stations around a liquid handling robot with an integrated arm. For imaging of plants during growth, an integrated robotic arm delivers EcoFAB 2.0 devices to imaging stations (**Figure 1A**) to collect scans and hyperspectral images of plant roots and shoots, respectively. By incorporating automated AI/ML analysis to estimate plant health and mass, this process can be integrated into Gaussian Process modelling and Bayesian Optimization to guide experimentation (**Figure 1B**).

### Testing and validation of the EcoBOT

Plant sterility was maintained during experimentation on the EcoBOT. Sterile seeds were germinated and loaded into autoclaved EcoFABs in a biosafety cabinet with sterile media and components. To initially assess the sterility of EcoBOT growth conditions, EcoFAB 2.0 devices with rich growth media (i.e., R2A or LB broth) were incubated on the robot for 2 weeks and then the devices were inspected for turbidity. None of these EcoFABs demonstrated any noticeable microbial growth. The EcoFABs used in all experiments were assessed through visual inspection and microbial growth assays from samples collected at harvest. Any EcoFABs that demonstrated positive growth were discarded from analysis. For example, there were a total of 142 *B. distachyon* plants grown on the EcoBOT for the various experiments discussed in this study. Of those, 126 were used in the final analyses and none of them were omitted due to contamination concerns. However, 3 plants died prematurely for reasons that were not investigated. Of the remaining 13 that were removed, 8 were in a single experiment where atypical, substantial root growth was observed outside the viewing window in the EcoFAB root chamber in one experiment and 5 were omitted due to issues with the imaging data.

Hydroponics were used in the EcoFAB devices on the EcoBOT for clearer root images. These conditions allow us to easily study nutrient and chemical stresses. As a first test, we examined whether *B. distachyon* had the expected phenotypic responses to nutrient stresses by growing plants in the EcoBOT in replicates of 4 for 30 days. Each plant was supplied with 50% Murashige and Skoog (MS) media (previous work determined that 50% MS media is replete for *B. distachyon*) with and without each of the core nutrients removed, or reduced individually. These components were: nitrogen (N; as ammonia and nitrate), potassium (K), phosphate (P), calcium chloride (CaCl_2_), magnesium sulfate (MgSO_4_) and ‘micronutrients’ which included the other inorganic constituents of the media. Specifically, the following individual modifications were tested (all relative to 50% MS media): removal of 50 or 100% of total nitrogen (½ N and No N), removal of 50 or 100% of potassium (½ K or No K), removal of 50 or 100% of phosphorus (½ P or No P), removal of MgSO_4_ (No MgSO_4_), CaCl2 (No CaCl_2_), or micronutrients (i.e., all compounds supplied in small amounts; No Micro).

**Figure 3:**
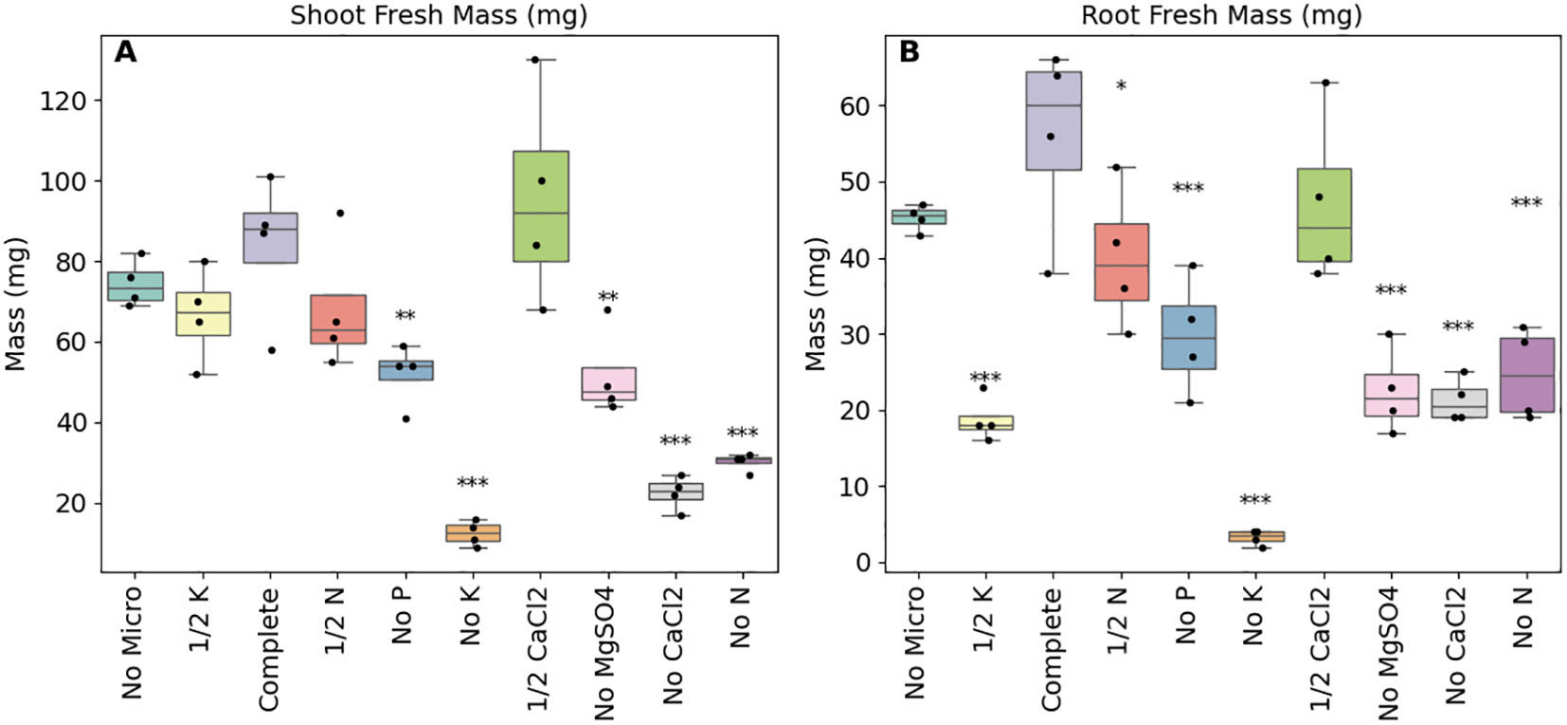
As expected the removal of essential nutrients resulted in the reduction of both shoot and root biomasses. Shoot **(A)** and root **(B)** fresh weights of *B. distachyon* under different nutrient stresses by altering the base media (50% MS) by either removing (‘No’) certain nutrients or by reducing the concentrations by half (‘½’), n = 4. Fresh weights were recorded at the end of the experiment and following ANOVA, pairwise comparisons (Tukey HSD) were made between the control media and the individual modified media. ***adjusted *p*-value (*p*-adj) of less than or equal to 0.001, **p-adj< 0.05, *p-adj< 0.1.

After 30 days, the plants were harvested and root and shoot fresh weights were recorded. In line with expectations, all of the complete nutrient knockouts, with the exception of the micronutrients free media, were significantly smaller than the control plants (*p*-adj<0.1; **Figure 3**). The complete removal of potassium, calcium or magnesium from the growth media had the largest impacts followed by nitrogen and phosphorus. Effect sizes for these treatments relative to the full nutrient controls ranged from 0.517 and -5.44 for shoot masses and between -0.725 and -5.83 for root masses (**Supplementary Table S2**).

### Bayesian optimization of sequential EcoBOT experiments enable quick copper stress assessments

We then decided to examine how Bayesian Optimization could be used with the EcoBOT to model plant responses to stressors. We selected copper in the form of CuSO_4_ as the stressor, since it is widely used for pest control and can accumulate in soils to levels that can inhibit plant growth and thus provides an important and tractable problem for initial EcoBOT experiments. A pilot experiment was conducted where *B. distachyon* plants were dosed with 500 **μ**M CuSO_4_ (or about 32 mg/L of Cu^2+^) after 10 days on the EcoBOT. This concentration was selected because we expected it to be inhibitory as it exceeded reported EC10 values (effective concentration that inhibits 10% of the population) for grasses in a study (see summarized data within Paschke et al) (Paschke and Redente, 2002)). After 14 days of growth post amendment, we confirmed significant inhibition of both root and shoot fresh biomass (**Supplementary Figure S5**).

Having confirmed the anticipated inhibition of copper on *B. distachyon* growth, we next examined the use of a range of AI/ML tools to: 1) process and analyze root and shoot images and 2) use Bayesian Optimization of experiments in combination with gpCAM analysis of results. gpCAM is a Python implementation of a Gaussian-Process-driven modeling package that has been used in autonomous experimentation at several experimental facilities. Bayesian Optimization is a well-established approach, and has been used with automated systems to drive experimental design and accelerate biological research (Radivojević et al., 2020). It has found widespread use across scientific research, but plant research has mostly focused on the selection of genotypes from field trials (Rincent et al., 2017; Tanaka and Iwata, 2018). To the best of our knowledge, this has not been attempted before for sterile plant phenotyping.

**Figure 4:**
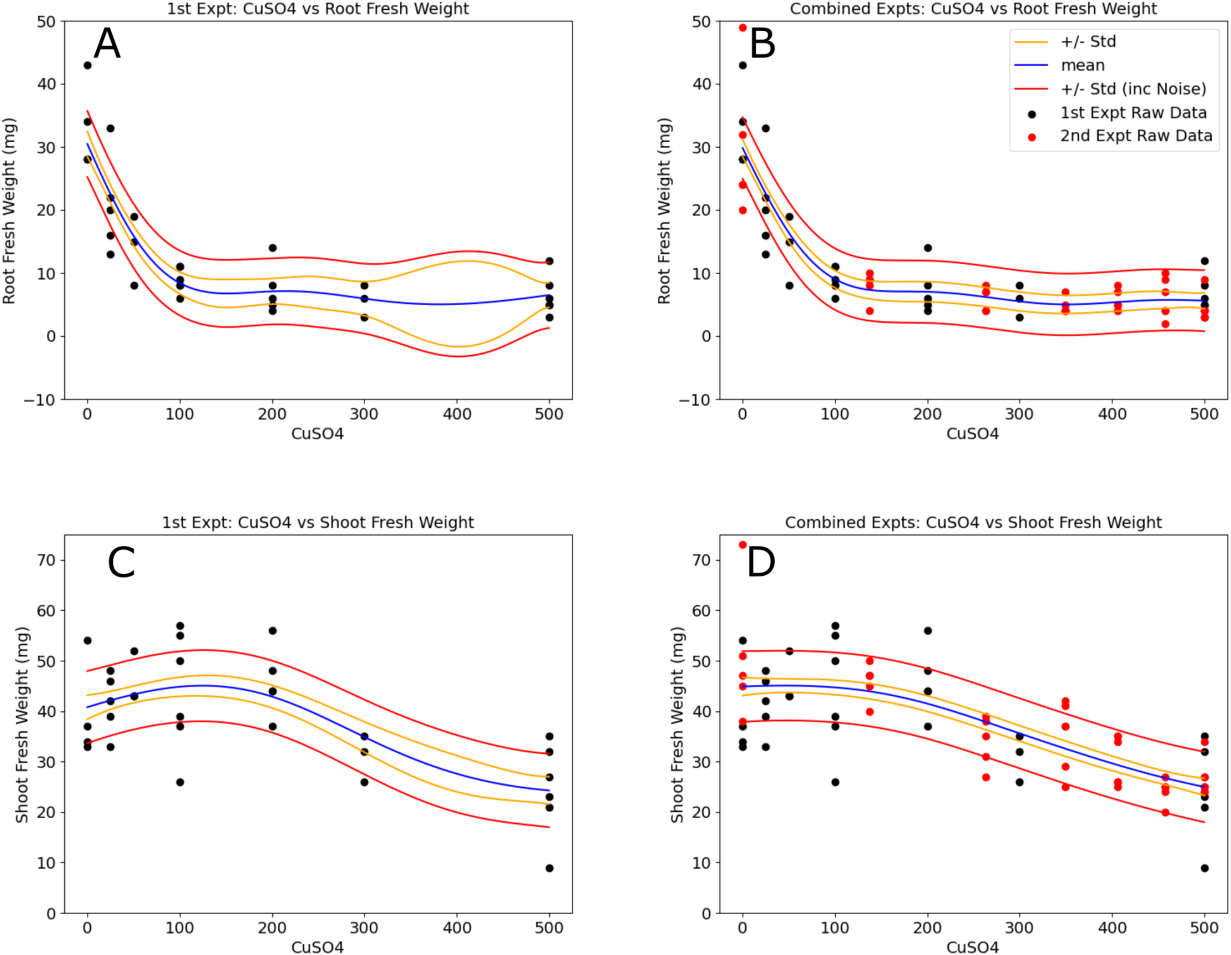
Models of copper vs *B. distachyon* root and shoot fresh weights improved with the incorporation of the second experimental datasets. Models in the left column **(A, C)** were made using the 1st experimental round. Regions in the concentration range where the relative variance is higher can be observed and correspond to the data added [red points in the right column plots **(B, D)**] from the 2nd experimental round. The resulting models have a much more uniform distribution in model standard deviations. Blue lines represent the model means, gold lines show one standard deviation of the model, and the red lines incorporate noise into the standard deviations.

To evaluate Bayesian Optimization workflow on the EcoBOT, we began by selecting 5 CuSO_4_ concentrations to test in between the two initial concentrations (0, 500 **μ**M). As with the pilot, we began by growing 40 *B. distachyon* plants in the EcoBOT for 10 days in 30% MS media, after which, water was replenished and amended with 1 of 7 additional concentrations of CuSO_4_ in replicates of 6 (0, 25, 50, 100, 200, 300 and 500 **μ**M final overall concentrations; **Figure 4**). The plants were grown for an additional two weeks without additional adjustment to the media and imaging of the roots and shoots occurring approximately every other day (**Supplementary Table S3**).

At harvest, root and shoot fresh weights were recorded for each plant; 10 of the 42 plants were removed from the study at this time (**Supplementary Table S4**), either due to issues with imaging or the development of an atypical phenotype where substantial, secondary root growth was observed outside of the root chamber. This was not observed in other experiments, but because these roots were outside of the root chamber, they could not be monitored in the same manner, and it is unclear what other effects this might have on the plant growth.

This fresh weight data was used to generate simple GP models (CuSO_4_ vs root, shoot and total fresh weights) and 5 additional CuSO_4_ concentrations were selected for a subsequent experiment where the initial CuSO_4_ vs root biomass model variance was the highest: 137, 263, 349, 406 and 458 **μ**M (**Figures 4B, D**, red points). These concentrations along with the 0 and 500 **μ**M conditions were used in the second round of experimentation. Of the 42 initial plants, 39 were kept for analysis. In total, these two experiments took 6 weeks of operating time on the EcoBOT and 1,633 root images and 4,899 hyperspectral images were collected and analyzed from the 71 plants left in the analysis.

**Figure 5:**
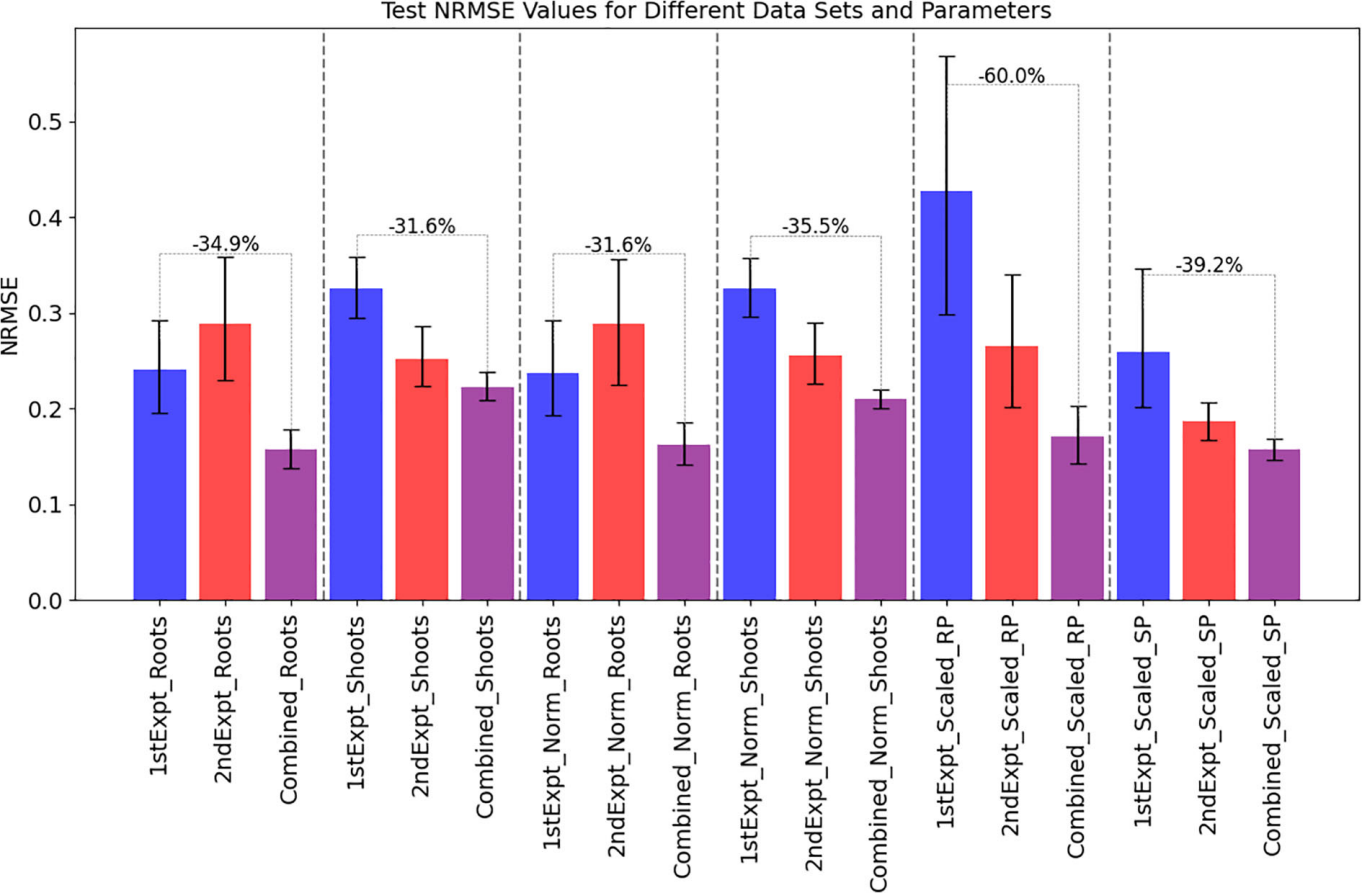
Model normalized root mean square error (NRMSE) values indicating predictive accuracy were routinely better when the data from the 2nd copper experiment were incorporated into the model. Models of added copper concentration vs root and shoot fresh weights (“_Roots”, “_Shoots”, sets 1 and 2) with and without normalization by MinMax (‘Norm_Roots’, ‘Norm_Shoots’, sets 3 and 4) scaling data within each experiment and using root and shoot pixel data MinMax scaled over the combined datasets (‘Scaled_RP’, ‘Scaled_SP’, sets 5 and 6) were used to make and evaluate Gaussian Process models. To evaluate model performances and improvement, 5 iterations of 6-fold cross validation were performed on each model (n=30). Blue, red and purple bars show the mean values of the model NRMSE test set predictions of the 1st experimental dataset, 2nd experimental dataset and the combined datasets, respectively. Black bars show 95% confidence intervals of the values and the annotated gray bars indicate the % improvement of the models between the 1st experimental model and the combined model.

Using data from the two experiments, we evaluated simple CuSO_4_ vs fresh weight models (**Figure 4**). It is evident by visual inspection alone that model variances were reduced by incorporating the second experimental dataset (**Figures 4A, C, B, D**). To evaluate the changes in the predictive power of the models before and after the second experiment, we compared the normalized root mean square error, or NRMSE values (**Figure 5**) of 30 test sets (5 iterations of 6-fold crossvalidation test train sets) for comparison (**Supplementary Data**). These were then used to calculate NRMSE values for GP models built for the individual and combined experimental data using either: 1) root and shoot fresh weights, 2) MinMax scaled root and shoot weight data ("Norm") or 3) pixel counts from the root ("RP") and shoot ("SP") images. In all cases, ANOVA analysis found RMSE values of real test data outperformed shuffled datasets (**Supplementary Table S5**).

For the root and shoot fresh weights, NRMSEs of the models decreased on average by 34.9% and 31.6%, respectively (**Figure 5**) when comparing the predictive performance of the first model’s to that of the combined model. Specifically, for plant roots and shoot fresh weights, the mean NRMSE test set values decreased from 0.24 (0.2 - 0.294, 95%CI) to 0.157 (0.138 - 0.178, 95% CI) and from 0.325 (0.296 - 0.356, 95%CI) to 0.222 (0.208 - 0.238, 95% CI), respectively (**Figure 5**, **Supplementary Data**). The combined model also produced lower NRMSE values than the 2nd experimental model (**Supplementary Figure S6**) and ANOVA analyses found these differences across the three models significant for each data type (p< 0.05; **Supplementary Tables S6, S7**) with the values significantly different between the 1st experimental model and combined model for root (p-adj< 0.1) and shoot (p-adj< 0.001). For the shoot models we found that the NRMSE values were significantly different between the two experiments (p-adj< 0.01) so we compared the root and shoot fresh weights for the control (0 **μ**M CuSO_4_) plants to look for potential batch effects. However, there was no indication that either the root or shoot distributions were significantly different between the two groups (p-values of 0.62 and 0.2, respectively for root and shoot fresh weights using Student’s T-Tests).

MinMax scaling is often used to normalize datasets prior to analysis in order to account for changes in their distributions. However, in this case, normalization by scaling each fresh weight dataset before incorporating them into the model did not significantly improve NRMSE values in any of the models (**Supplementary Data**). Therefore, only MinMax scaling across all data was used in subsequent analyses. In particular this was used with pixel count data as described below to keep length scales more manageable.

There were also significant differences in the monitored temperatures (**Supplementary Tables S8, S9** as well as **Figure 2** (1st Copper)) between the first and second experimental rounds so we also evaluated whether incorporating the environmental parameters (i.e., light intensities and average root and shoot day/night temperatures, **Supplementary Data**) improved the NRMSE values, however, there was no significant change in the values (**Supplementary Table S10**).

A limitation of the above analysis with root and shoot fresh weights is that these were only collected at harvest. While this is a very standard approach in plant research, the EcoBOT also generated time-series images of the roots and shoots. To explore the relationship between the imaging data and weight measurements, we compared the images and weights collected on the final day of the experiment. For roots, root pixels from RhizoNET segmentation were used and for shoots the Euclidean Normalized counts from the 3 angles produced through EcoSpec segmentation were used. This approach has been evaluated before (Poiré et al., 2014) and in this case the Euclidean Normalized data fit as well or better than summing all counts or using individual angles (**Supplementary Table S11**). For simplicity, the Euclidean Normalized counts are referred to simply as *pixel counts* hereafter.

For both root and shoot datasets, these pixel counts were MinMax scaled over the course of both experiments for all analyses. Strong correlation values were observed in both cases with the Pearson correlation coefficient between root pixels and root fresh weights of 0.87 (0.80 - 0.92, 95%CI) and 0.85 (0.77 - 0.91, 95%CI) for the shoot pixels and fresh weights (**Supplementary Figure S7**). While these strongly correlate and are of predictive value, we will refer to the size information generated from the images as relative plant size estimates or scaled root (RP) or shoot (SP) pixel counts here.

To determine how well these models performed using non-invasive imaging data instead of destructive weight data, we MinMax scaled the pixel count data (scaled-RP, scaled-SP) over both experiments and remade the models. Model performances again demonstrated a decrease in NRMSE values of 60% and 39.2% for root and shoot measurements, respectively (**Figure 5**). Using these metrics the scaled root pixel model NRMSE measurements decreased from 0.427 (0.299 - 0.594, 95%CI) to 0.171 (0.142 - 0.202, 95%CI) and from 0.258 (0.2 - 0.356, 95%CI) to 0.157 (0.148 - 0.167, 95%CI). This indicated that using the imaging for further analyses would be informative.

### Time-series responses of *B. Distachyon* phenotypes to copper exposure

The *B. distachyon* imaging data allowed us to evaluate phenotypic responses over time and include health estimates. Root and shoot images were collected approximately every other day in both experiments (**Supplementary Table S3**). Initial shoot health estimates were calculated by performing UMAP deconvolution within EcoSpec, separating the pixels into a healthy and unhealthy bin and determining the fraction of shoot pixels assigned to the ‘healthy bin’. In order to get daily estimates all imaging data were smoothed with a Savitzky-Golay filter (Savitzky and Golay, 1964) and then interpolated to get daily estimates from days 4 to 25.

**Figure 6:**
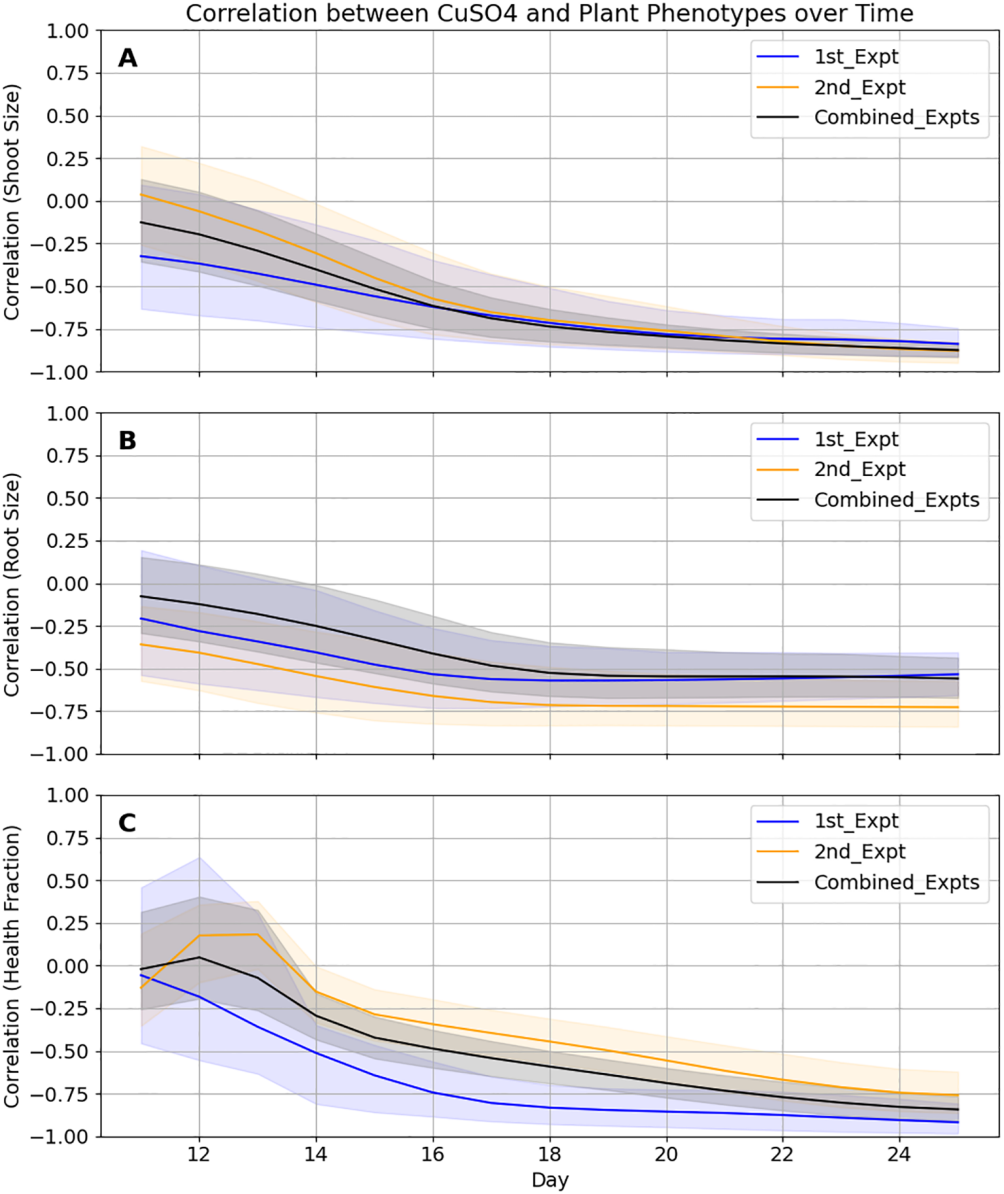
Correlations between *B. distachyon* phenotypes and CuSO4 concentrations become more accurate when the two datasets are combined and converge at different rates. In all cases, 95% confidence intervals (shaded areas) for the Pearson Correlation coefficients for the 3 phenotypes are tighter when the data from the two experiments are combined. **(A)** While the values for roots appear to be finalized a week before harvest, **(B)** shoot size values are still changing up until a few days before harvest and **(C)** health values may change still if the experiment was extended.

Pearson correlation analyses at each time point between added CuSO_4_ concentration and the estimated root size, estimated shoot size and shoot health provided a quick way to initially evaluate these relationships. Also, it provided additional information on how incorporating the 2nd experiment clarifies these relationships. Following the first experiment, mean correlations between CuSO_4_ and estimated root size, estimated shoot sizes and shoot health at the end of the experiment were -0.53, -0.84 and -0.92 with 95% confidence intervals of (-0.66, -0.40), (-0.91,-0.74) and (-0.98, -0.81), respectively (**Figure 6**). Incorporation of the second experiment changed those values to -0.56, -0.88 and -0.84 and narrowed the ranges of the confidence intervals by between 8% and 50%.

**Figure 7:**
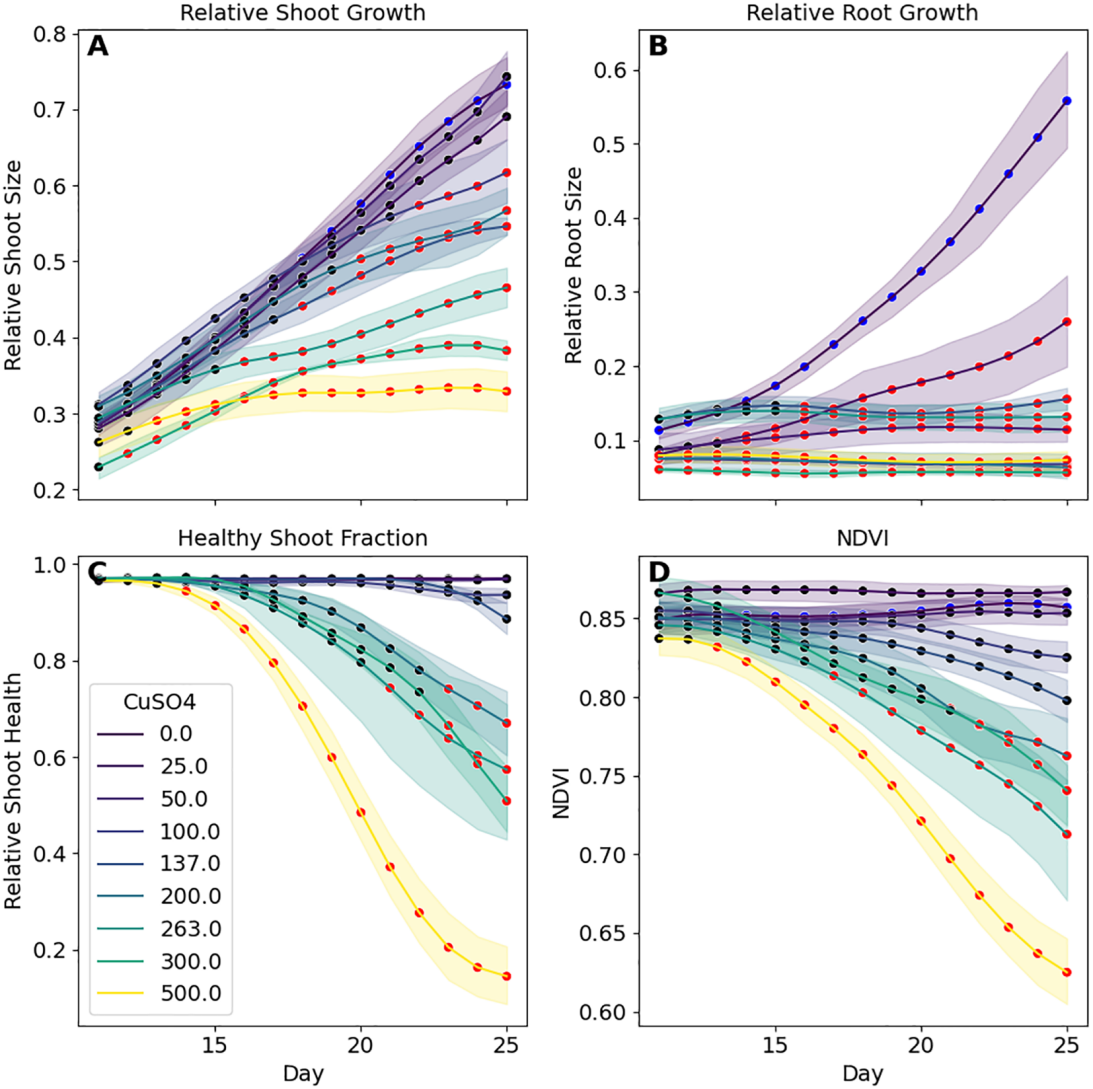
Time series analysis of *B. distachyon* phenotypic responses to CuSO4 concentrations shows that sensitivity varies based on the specific metric and length of time. **(A)** Significant differences (red markers; *p*-adj< 0.05) in shoot sizes are not observed until plants were exposed to over 200 **μ**M of CuSO4 whereas differences in **(B)** roots were observed at 25 **μ**M. Changes to shoot health metrics **(C, D)** required even higher CuSO4 concentrations and were slower to show than in plant sizes. Each datapoint is the mean with shaded regions representing 95% confidence intervals.

To get more detailed information on these combined datasets, we performed statistical comparisons between each tested CuSO_4_ concentration post amendment (i.e., Days 11 - 25). A Normalized Difference Vegetation Index (NDVI) value (Bannari et al., 1995; Zwart et al., 2025), a metric that can be used to assess plant health and density, was also calculated for each plant using the hyperspectral data. Multicomparison testing between each concentration and the control plants for relative root and shoot sizes, shoot health fraction and NDVI were conducted for each day (**Figure 7**). Investigation of the root images revealed that even at 25 **μ**M CuSO4 there was significant inhibition in root growth after 3 days, with significant differences observed almost immediately at 500 **μ**M. Shoot size was less sensitive to CuSO_4_ concentrations. 100 **μ**M of CuSO4 was necessary for impacts to be significant and at that concentration impacts weren’t significant until 12 days post addition. As with the roots, differences were observed faster at 500 **μ**M, 5 days, but still lagged behind the observations in the roots. Shoot health was the least sensitive with significant loss of color not observed in the control plants until 6 days (at 500 **μ**M) and impacts weren’t observed until at least 200 **μ**M addition.

**Figure 8:**
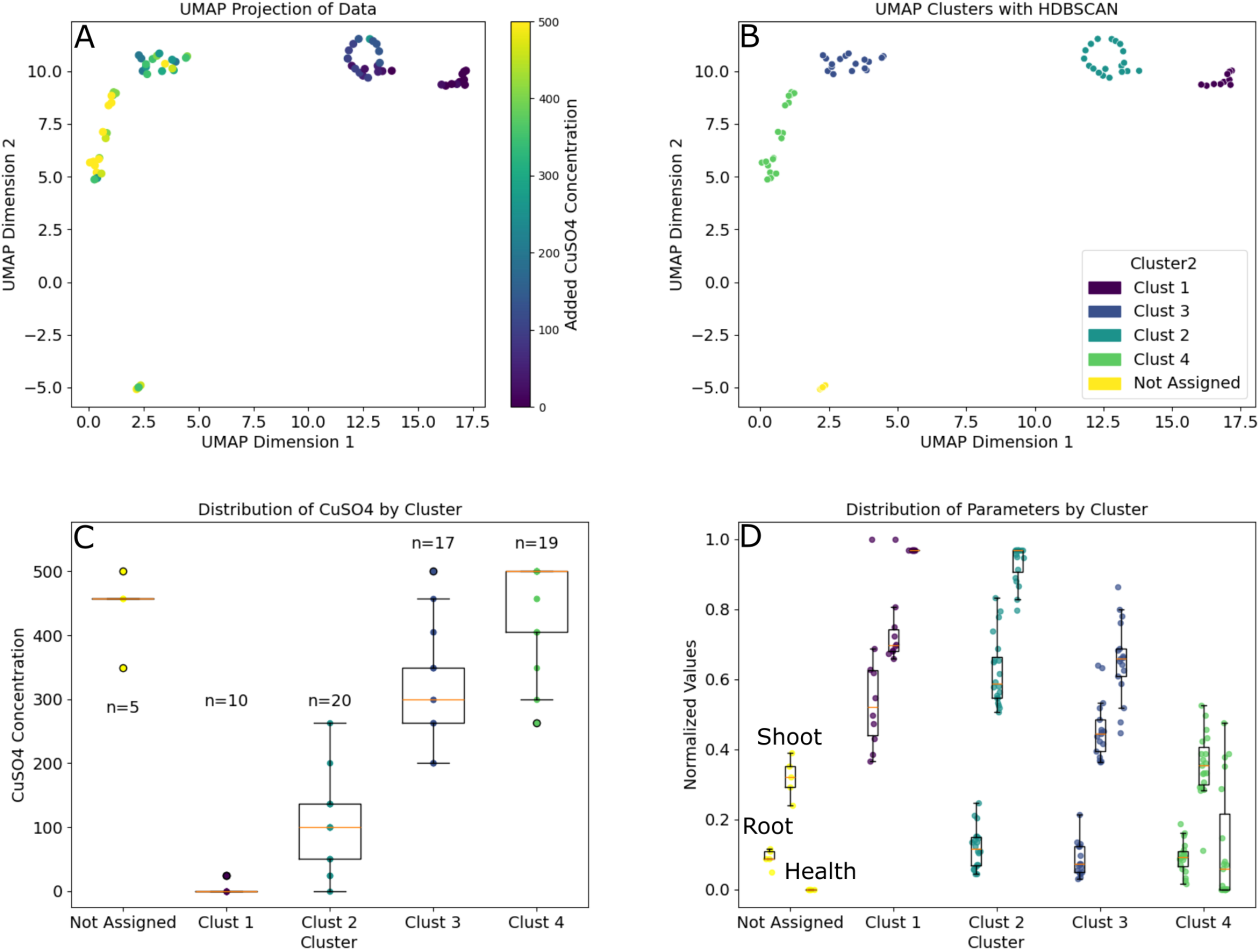
Time series data clustered using unsupervised learning to show how the concentration of the CuSO_4_ amendment impacts various *B. distachyon* phenotypes. **(A)** 2D projection of the time-series estimated root and shoot sizes combined with health fraction data from the two experiments from the time CuSO_4_ was added to the plants until harvest. **(B)** HDBSCAN clustering of the projection in A where 66 of the 71 plants were assigned one of 4 clusters. **(C)** Distribution of CuSO4 concentrations for the plants in each cluster along with population sizes for each group. Note: Clusters 1–4 were all significantly different from one another (*p*-adj< 0.05). **(D)** Distribution of final root and shoot sizes and health fraction (left to right in each cluster) data for each group. Root sizes in Cluster 1 were significantly larger than all other clusters, all shoot sizes were significantly different from one another, and health fractions were significantly different between all clusters except between Clusters 1 and 2.

Finally, we used a hybrid unsupervised learning approach to examine root and shoot responses to the amended copper. We took the time-series data from days 11 to 25 for the root and shoot size estimates along with the shoot health fraction data and projected these data in UMAP (**Figure 8**). Examining the clustered data (HDBSCAN) resulted in 66 of the 71 analyzed plants falling within 4 clusters corresponding with significantly different (*p*-adj< 0.01) average CuSO_4_ concentrations of 5, 99, 315 and 445 **μ**M. Examination of the three phenotypes on the final day found that these clusters represent significant differences in these metrics. Final root size estimates were significantly larger at the lowest concentration than all other clusters. Shoot size estimates were significantly different between all clusters (*p*-adj< 0.05) and health fractions in the 315 and 445 **μ**M clusters were significantly smaller than the clusters centered around lower concentrations (*p*-adj< 0.001).

Thus, through these analyses of the EcoBOT’s time-series data over the course of these two experiments, we obtained information on both the time frames and concentration ranges where significant phenotypic effects were observed in *B. distachyon*. Specifically, we found specific windows of impact for CuSO_4_ inhibition that vary based on concentration and phenotype of interest (**Figures 6**-**8**) and then used that information to find specific ranges of copper where specific combinations of *B. distachyon* phenotypes were impacted.

## Discussion

### Construction of the EcoBOT from conventional automation and imaging equipment

The EcoBOT was designed to automate root and shoot phenotyping of sterile, small model plants using commercially available equipment where possible. *B. distachyon* was used in this study, however other plants that can be maintained in the 50 mm tall growth chamber and the 10 ml root chamber over the course of an experiment could be used. The growth chamber has 3 shelves and each has up to 57 positions for EcoFABs that are accessible by the robotic arm allowing for experiments of over 150 plants at time. However, this could be expanded by reorienting the rear cabinet and installing a second chamber to potentially double the capacity. To minimize the overall footprint and provide necessary functionality without designing a new system from scratch, it is built around a liquid handling system with a robotic arm that can access all stations in the system (**Figure 1**). When closed, the entire system including the growth chamber, imaging stations and liquid handling unit fits within a 4 m^2^ area making it suitable for laboratories. Integrating and automating the operation of these units was one of the more challenging aspects of building the system as they are not designed to work with scripts and lack APIs, so where command line codes could not be used, interactive macros are used to operate the equipment unsupervised. In addition, for the hyperspectral camera, three different motion systems needed to be designed and/or incorporated to generate images from multiple angles. The selected turntable was already compatible with the EcoBOTs control software, however two actuators were designed and mounted for this system specifically: 1) to turn on the camera as a physical power button needs to be pressed and 2) to raise and lower the camera to get images from the sides and top of the EcoFABs. In theory, dedicated PLCs (programmable logic controllers) could be designed to make these operations more robust and less susceptible to interruption. Videos demonstrating the EcoBOT can be found in **Supplementary Data**.

### Experimental design and sources of variability

Experimental consistency is a goal in plant phenotyping platforms to allow integration of datasets across multiple experiments. This can be particularly important due to the inherent, biologically-driven variability in these experiments. The use of EcoFABs aids this in part by maintaining a sterile, or user-defined, environment for plant growth. Consistent with previous EcoFAB 2.0 studies (Novak et al., 2024a; Novak et al., 2024b), contamination was not observed in our EcoBOT experiments. However, due to the compact nature of the system, there were design constraints which led to heterogeneity in light intensity and speciation. In addition, while the system has HEPA filtered airflow and conductive temperature control via heat exchangers, there were small, but significant differences in temperature between experimental rounds (**Figure 2**; **Supplementary Table S9**). However, the described nutrient evaluation experiment demonstrated (**Figure 3**) that the removal of key metabolites from the growth media significantly reduced *B. distachyon* biomass in EcoFABs in the EcoBOT chamber and there were not statistically significant batch effects observed between experimental rounds in the copper experiment.

That being said, as with all experiments increased replication will improve reliability of findings. For our initial experiments only 4 replicates were used, informed in part on the initial EcoFAB 2.0 study (Novak et al., 2024a) which suggested that 5 EcoFABs were sufficient for nitrogen speciation studies. Indeed, we found that in several cases, as few as 4 plants were adequate for comparative analyses (**Figure 3**), however, there were some interventions that did not reach statistical significance at this replication level. With this in mind, we increased replication to 6 for the copper experiments. While this was appropriate for the high concentration CuSO_4_ comparisons used in the pilot test, more detailed assessments would benefit from even higher replication and would help account for unexpected outliers as were observed in the first round of copper experiments.

### Successful application of Bayesian optimization

We performed what is to our knowledge, the first Bayesian Optimization experimentation on sterile model plants; focusing on the impacts of copper stress on plant phenotypes. While this strategy has been used in plant breeding, we believe there are exciting opportunities to use this strategy for understanding many aspects of plant growth in combination with a system like the EcoBOT. In particular, in this study, we performed a very simple pair of sequential experiments to explore the phenotypic impacts of copper on *B. distachyon.* To this end, we used draft models from the 1st experiment to inform the design of the 2nd experiment by selecting conditions most likely to improve the confidence of the GP model predictions.

The additional data improved the confidence in the model fits, and the predictive power with NRMSE values improving by >30% for the fresh weight models (**Figure 5**; **Supplementary Figure S6**; **Supplementary Data**). Incorporation of other environmental variables measured, i.e. temperatures and lighting, did not significantly impact model prediction NRMSE values.

It is important to note that while we employed a Gaussian Process Bayesian Optimization strategy to identify experimental conditions that would best to reduce model uncertainty, alternative strategies could have been used effectively in this study. Previous research (Noack et al., 2019) has shown that when working with sparse datasets, and limited experimental cycles, Bayesian Optimization does not always hold a significant advantage over other methods including random sampling, however it can not perform worse. Therefore, in this particular case, while our Bayesian Optimization approach was able to quickly improve these models, other methods would likely have worked comparably in this instance.

### Time is an important variable to consider in plant phenotyping

Analyzing the impacts of the stressor, copper, on *B. distachyon* phenotypes was also conducted over time. We monitored root and shoot sizes as well as plant health proxies for each plant via images from before copper was added until harvest. We learned that not only are each of these phenotypes impacted differently by given copper concentrations, but the rate of onset of these effects varied between them (**Figures 6**-**8**). In general, the time for deleterious phenotypes to be observed was inversely related to the concentration of copper (**Figure 7**). However, the speed at which these changes occurred varied by phenotypic measure.

Understanding this information could impact future experimental designs in several ways. For example, the impact on root growth at the highest tested concentrations was almost immediate and overall, correlation between copper concentration and root biomass was established about a week prior to the harvest (**Figure 8**). Therefore, studies solely concerned with root health could be shortened, allowing for faster turnaround. Similarly, studies evaluating intervention strategies to mitigate these impacts may need to be applied immediately after exposure. Conversely, studies on the impacts in shoot health may need to be longer to fully appreciate impacts since the correlation values never flattened over the course of this particular study (**Figure 6**). If a causal relationship was sought-after between copper concentrations, root growth and shoot growth/health, various timeframes would need to be evaluated and tested.

The time series datasets also helped to identify key concentration ranges of interest for future studies through unsupervised learning. When we incorporated the shoot and root sizes along with shoot health over the 2 weeks of copper exposure into a UMAP projection we found that the plants clustered into four groups with significant differences in the average copper concentrations and phenotypic observations (**Figure 8**). The plants in these clusters were exposed to average concentrations of 445, 315, 99 and 5 **μ**M of CuSO_4_, respectively (**Supplementary Data**). These concentrations provide information on where we see significant changes in *B. distachyon* phenotypes under the conditions and timeframes tested here.

### Biological observations and context

Excessive copper is known to impact plant growth in several ways including inhibited growth, cellular and photosynthetic damage, and disruption of nutrient uptake which have primarily been associated with increased oxidative stress (Paschke and Redente, 2002; Thounaojam et al., 2012). Consistent with previous studies, deleterious phenotypes were first observed in the root biomass before plant shoot biomass or health were impacted (Paschke and Redente, 2002; Thounaojam et al., 2012). This has been attributed to the fact that roots tend to accumulate more copper than shoots and are the primary site of copper-induced oxidative damage (Paschke and Redente, 2002; Thounaojam et al., 2012).

However, the design of this study likely contributes to the specific concentration ranges where these phenotypic changes were observed, which are lower than those reported in wheat and grasses which were grown in greenhouses in sand (Paschke and Redente, 2002). Two particular factors are that these experiments were performed hydroponically and that the roots were exposed to growth lights due to the transparent EcoFAB base. In hydroponic growth media, metals tend to be more bioavailable and are therefore capable of more relative accumulation and oxidative damage (Paschke and Redente, 2002; van der Ent et al., 2024) and it has been suggested that concentrations of copper in hydroponic media as low as 1 **μ**M likely has toxic impacts on plants (van der Ent et al., 2024).

Roots exposed to lights has itself been a documented source of stress that can include imbalanced reactive oxygen species (ROS) and hormone production as well as stunted root growth among other effects discussed by Cabrera et al (Cabrera et al., 2022). This can cause the copper induced stress to be compounded where plants are less able to defend against oxidative stressors and imbalanced nutrient uptake (Cabrera et al., 2022). Despite these caveats, while the data generated from this system might not translate directly to field observations, it has the potential to serve as a valuable tool for identifying and understanding the specific biological responses to various stressors and evaluate potential mitigation strategies under defined conditions. Furthermore, this work lays the groundwork for future research aimed at refining experimental design and improving predictive modeling in plant responses to environmental stressors.

## Limitations

There are a number of limitations with the EcoBOT and with the study presented here in particular that need to be considered. As mentioned above, *B. distachyon* were grown under hydroponic conditions in clear EcoFAB 2.0 devices so that root growth could be monitored and the base of the EcoFAB 2.0 devices (the component that separates the root and shoot compartments) was clear allowing roots to be exposed to the light. In addition for automation reasons, the EcoFAB 2.0 devices were incubated flat so there was little to no gravitropism. Future studies could potentially use sand or even soil, though this would complicate root imaging and EcoFABs with opaque bases could also address the light exposure to the roots, however further investigation will be needed. In addition, while we did monitor temperature in the system using probes in and outside of dedicated EcoFABs (**Figure 2**; **Supplementary Tables S5, S6**) these did not have plants growing in them which could lead to errors in our estimates of temperature and humidity profiles vs. the planted EcoFABs. Efforts are underway to make these predictions more accurate.

Finally, our analysis here is admittedly limited in scope. There are several plant phenotypic measurements that are common for plant phenomics that we want to include for future analyses beyond root and shoot masses including lengths, root branching, leaf counts, as well as a variety of hyperspectral analyses. However, the appropriate methods to incorporate these metrics into our analyses are still being explored and have not been incorporated into the automated software at this time. VIS-NIR hyperspectral imaging in particular has the power to monitor a number of plant health parameters, however, in this study we focused on a specific NDVI measurement for a couple of reasons. One was a practical limitation, as the lighting used for these particular experiments did not provide stable illumination beyond 880 nm, which limited the number of applicable, published hyperspectral indices that could be applied. The second was that previous analysis of this specific index taken of plants in EcoFABs found that its values corresponded well to visual interrogations of RGB images (Zwart et al., 2025). Thus upgrades to the lighting used in the imaging and more exhaustive benchmarking will improve future datasets.

## Future directions for the EcoBOT

This study demonstrates much of the potential as well as some of the limitations in using AI/ML in combination with automated plant phenotyping. Systems like the EcoBOT can accelerate research of model plants through the integration of non-invasive, automated imaging, AI/ML tools to extract phenotypic information from the images, data modelling using Gaussian Processes and experimental design through Bayesian Optimization. We envision that systems like the EcoBOT can be extended to other types of plant stresses and eventually be useful for studying how microbes mitigate these stresses. An advantage of conducting these experiments on the EcoBOT is that because the data recording, including meta-data, is standardized to make comparisons between experiments easier. This includes retroactively analyzing datasets as methods evolve. For example, these analyses were confined to root and shoot size estimates and a couple of spectral analyses for the shoots. As datasets expand and methods are refined these data could later either be incorporated into larger meta-analyses, or evaluated for additional information on root lengths, leaf counts or other phenotypic parameters of interest without much effort once those capabilities are in hand.

## Data Availability

The original contributions presented in the study are included in the article/**Supplementary Material**. Further inquiries can be directed to the corresponding authors.
