# Supplementary material for: EcoBOT: an AI/ML enabled automated phenotyping capability for model plants
Source: Front Plant Sci. 2025 Dec 2;16:1633557. doi: 10.3389/fpls.2025.1633557 (PMC12705611; doi:10.3389/fpls.2025.1633557)
Supplement: Supplementary file 1 [file DataSheet1.docx]

Supplementary Materials for

**EcoBOT: an AI/ML enabled automated phenotyping capability for model plants**

Peter F. Andeer*, *et al.*

*Corresponding author. Email: [pfandeer@lbl.gov,](mailto:pfandeer@lbl.gov,) trnorthen@lbl.gov

**This PDF file includes:**

Figures S1 to S11

Tables S1 to S7

Appendix S1 – Description of raw and processed data included in separate file

**Supplementary Text**

**
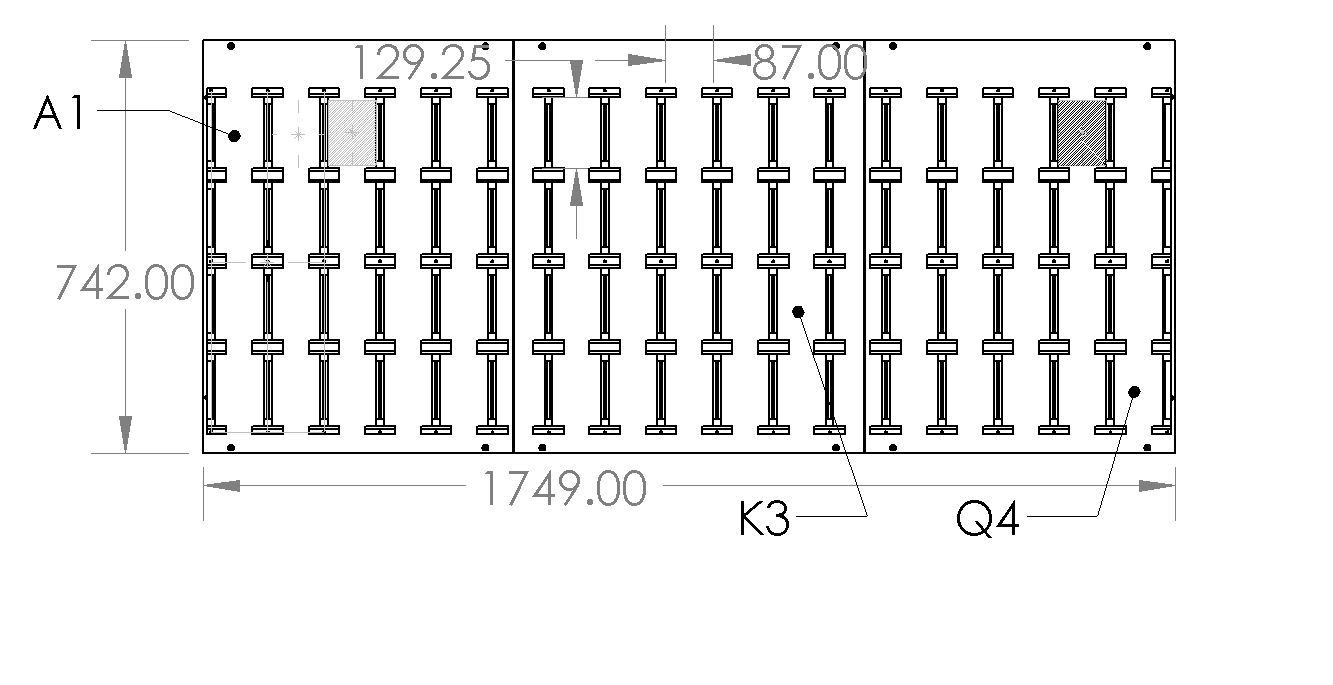
**

**Figure S1. General schematic of shelves in the EcoBOT growth chamber.** The shelves are set up in grids with rows labeled 1 - 4 and columns labeled alphabetically from A - Q. Shaded spaces (C1, P1) indicate locations of temperature monitoring EcoFABs. 3 locations are labeled for orientation purposes and general dimensions are listed for spaces and shelving. All measurements are in millimeters.

**
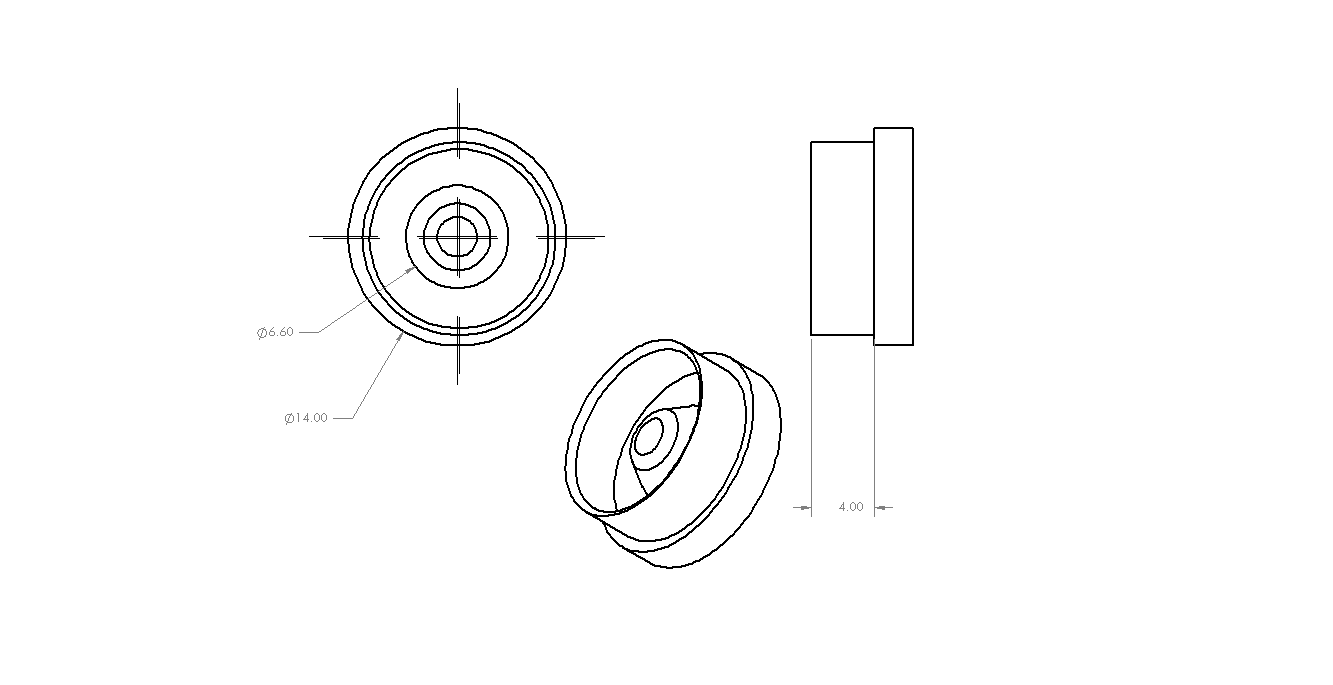
**

**Figure S2. 3D printed caps designed for the EcoFAB 2.0 to make it compatible with automation.** Caps are printed out of biomedical-grade resin (FormLabs BioMed Black, RS-CFG-BMBL-01, or Amber,RS-CFG-BMAM-01 ) and can be sterilized via autoclave. The flat top makes it possible for the EcoBOT to remove them using a suction cup tool. All measurements are in millimeters.

**
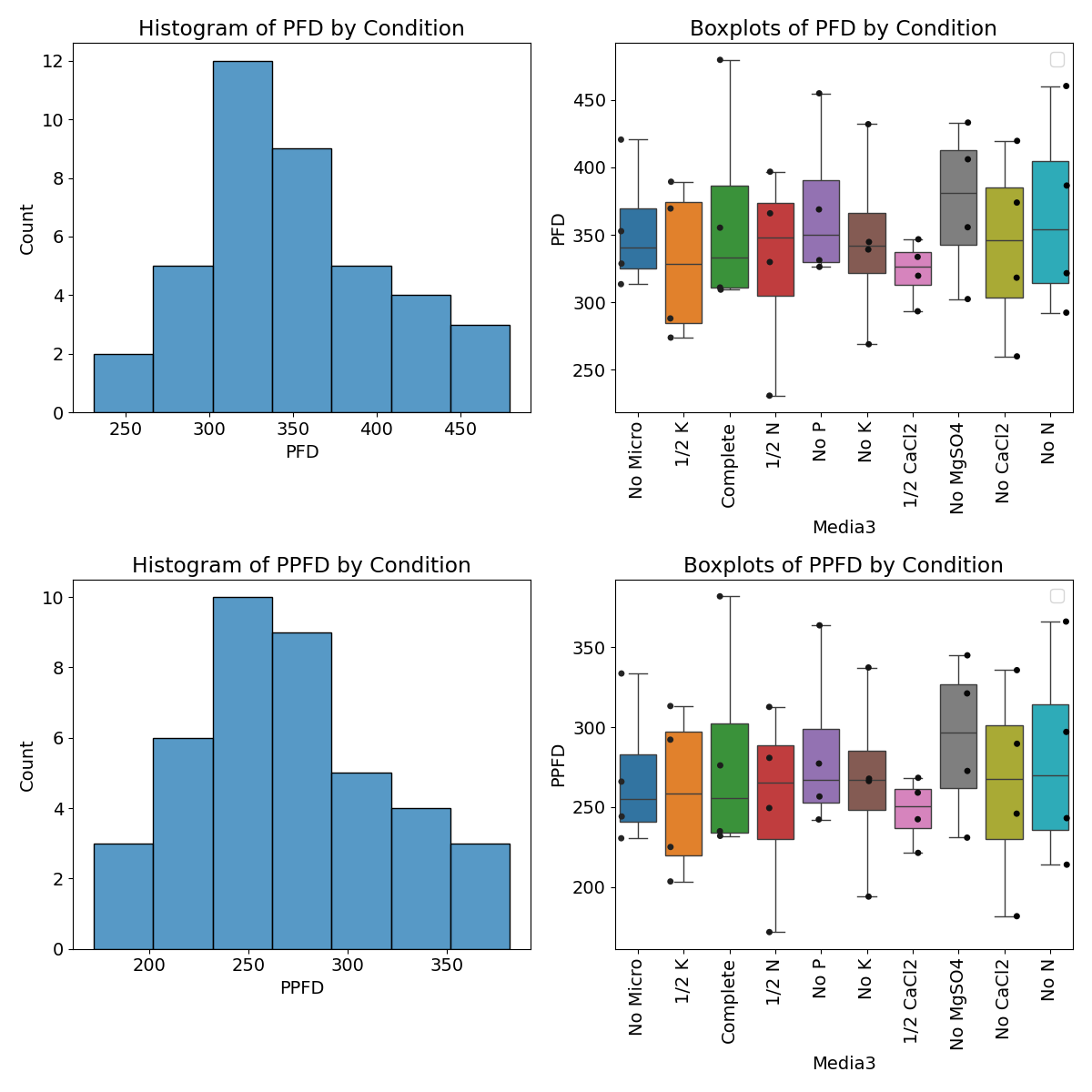
**

**Figure S3. Boxplot of estimated light intensities for nutrient stress experiment.** Light intensities for each spectra for the nutrient stress experiment were estimated using curves generated from multiple measurements using a LI-COR LI-180 spectrometer within each shelf location with each of the LEDs independently varied from 0 - 100% intensity. A 14 hr light cycle with an average PFD light intensity of 350 μmol/m²/s was used (Fig. S3., Note: light intensities for this experiment alone were based on standard curves made for each position and do not take into account the EcoFAB 2.0 chamber).


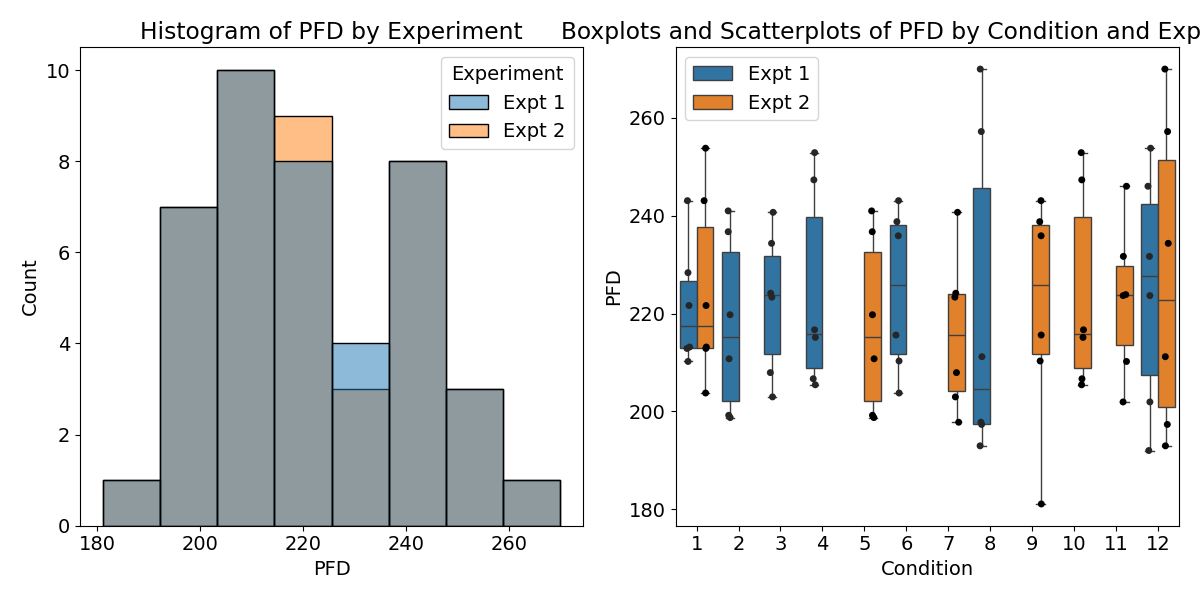


**Figure S4. Light intensity variation and distribution between treatment replicates can be tracked and accounted for in experiments.** Each of the two experiments used 42 EcoFABs distributed amongst the 56 possible locations on the top shelf of the EcoBOT. Average light intensity for the shelf was set at 222 μmol/m²/s PFD for the system and measurements used were acquired using a spectrophotometer mounted on a multiwell plate, placed in each specified shelf location being used and covered with an EcoFAB 2.0 chamber. The left hand side shows the overall distribution of intensities in the locations used for each experiment while the right hand side shows specific distributions within each treatment.


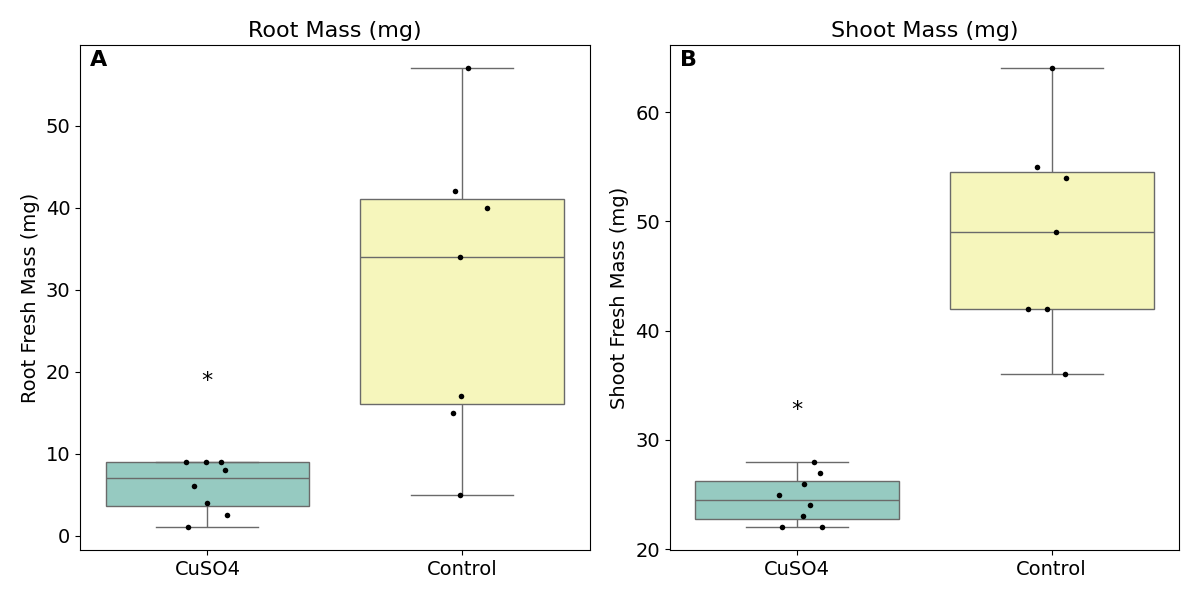


**Figure S5.  *B. distachyon* dosed with 500** 𝛍**M CuSO4 had significantly lower root and shoot fresh weights.** Plants grown on the EcoBOT for 10 days were dosed with media with and without CuSO4 and fresh weights were measured after an additional 14 days. * - *p*-adj < 0.01. Specific  *p*-adj values for root and shoot biomasses are 0.003 and 9.95e-6, respectively


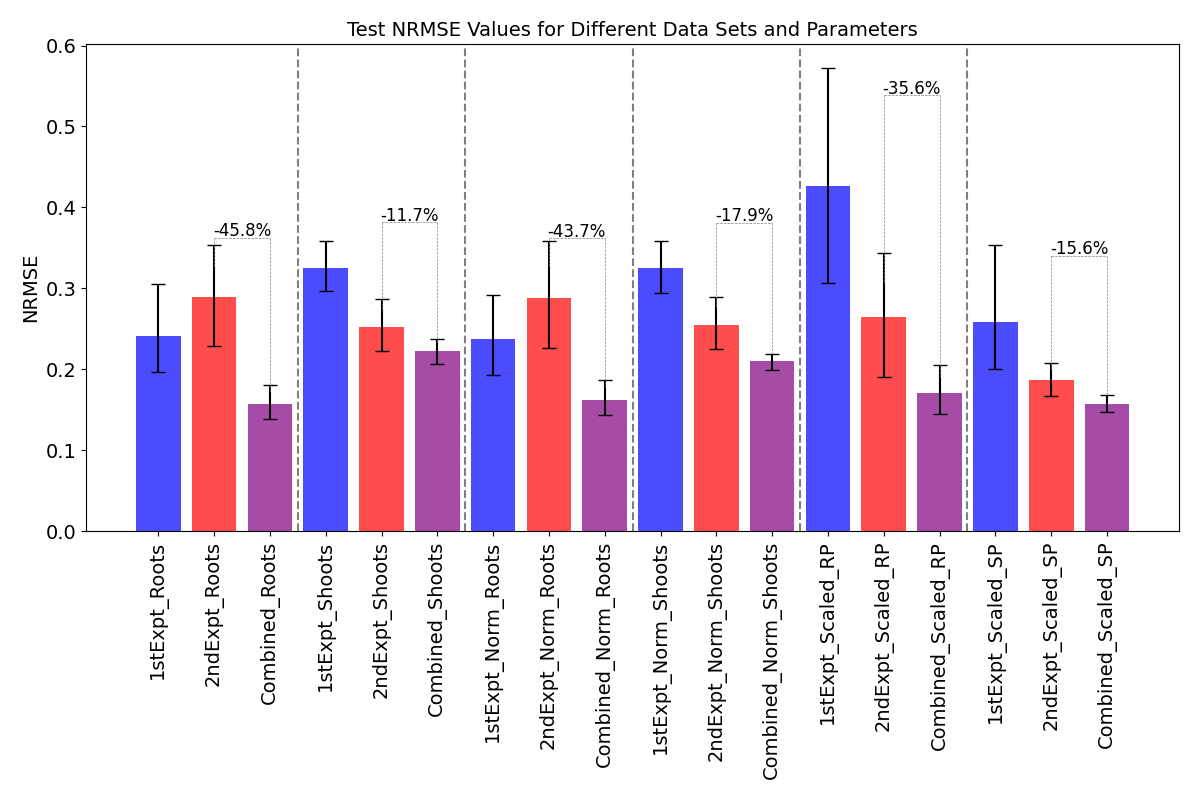


**Figure S6.** Model normalized root mean square error (NRMSE) values indicating predictive accuracy were routinely better when the data from the 2nd copper experiment were incorporated into the model. Models of added copper concentration vs root and shoot fresh weights (“_Roots”, “_Shoots”, sets 1 and 2) with and without normalization by MinMax (‘Norm_Roots’, ‘Norm_Shoots’, sets 3 and 4) scaling data within each experiment and using root and shoot pixel data MinMax scaled over the combined datasets (‘Scaled_RP’, ‘Scaled_SP’, sets 5 and 6) were used to make and evaluate Gaussian Process models to evaluate model performance and improvement using 5 iterations of 6-fold cross validation (n = 30). Blue, red and purple bars show the mean values of the model NRMSE test set predictions of the 1st experimental dataset, 2nd experimental dataset and the combined datasets, respectively. Black bars show 95% confidence intervals of the values and the annotated gray bars indicate the % improvement of the models between the 2nd experimental model and the combined model.


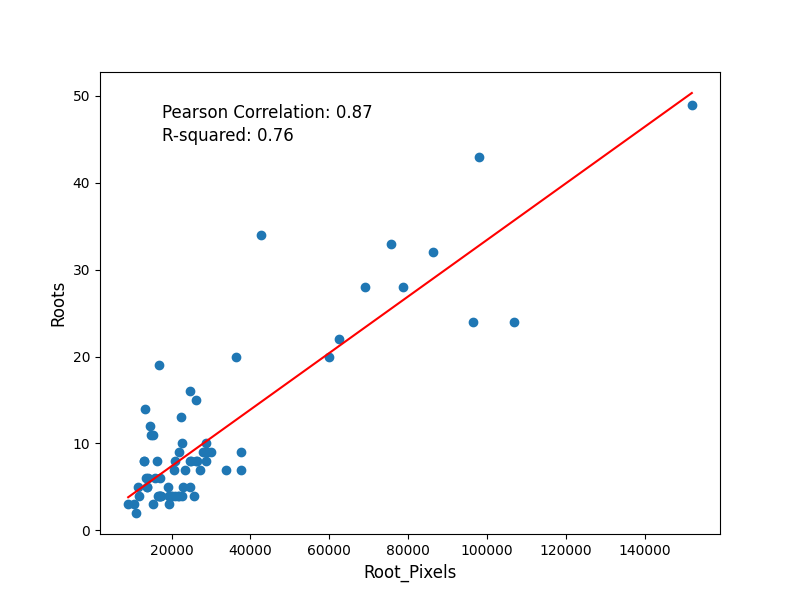

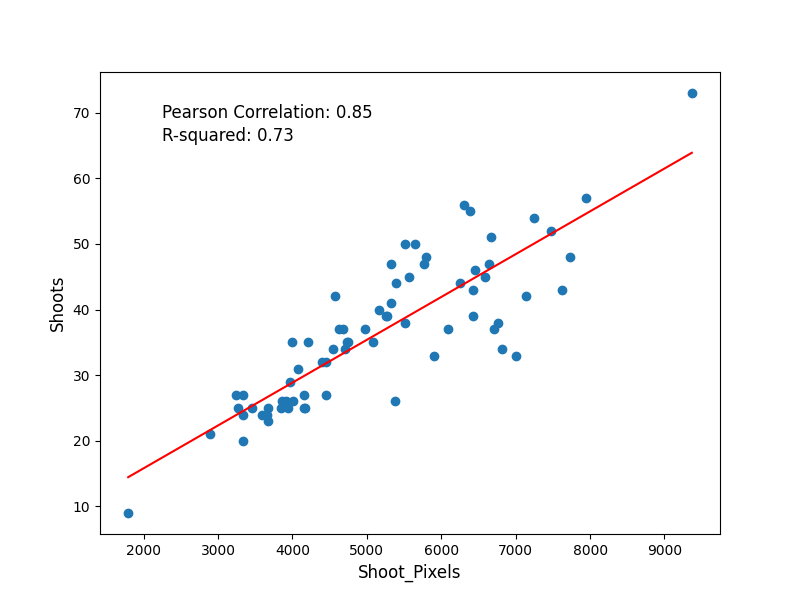


**Figure S7. Root and Shoot imaging data strongly correlates with respective fresh weights.** The left plot compares the raw pixel counts to recorded fresh weights for roots and on the right the Euclidean Normalization of the pixels counts for each of the 3 angles are compared to shoot fresh weights. The 95% confidence intervals for the Pearson correlation coefficients are (left, right): 0.80 - 0.92, 0.77 - 0.91.

**Table S1 Compositional breakdown of media used in the nutrient stress experiment**


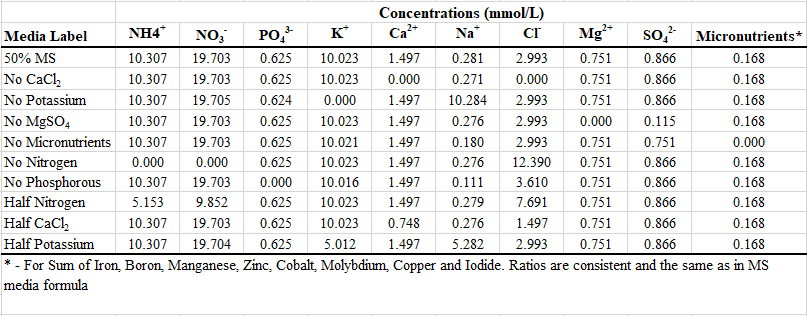


**Table S2. Effect Sizes of Nutrient Experiment Data**

|  | **Shoot Mass (mg)** | **Root Mass (mg)** | **Total** |
| --- | --- | --- | --- |
| **No Micro** | -0.683 | -1.181 | -0.925 |
| **1/2 K** | -1.111 | -4.022 | -2.407 |
| **1/2 N** | -0.895 | -1.429 | -1.131 |
| **No P** | -2.267 | -2.498 | -2.452 |
| **No K** | -5.444 | -5.833 | -5.741 |
| **1/2 CaCl2** | 0.517 | -0.725 | 0.088 |
| **No MgSO4** | -2.123 | -3.404 | -2.691 |
| **No CaCl2** | -4.626 | -3.759 | -4.384 |
| **No N** | -4.116 | -3.123 | -3.831 |

**Table S3. Timeline for the two-part copper experiment**

| **Action** | **1st Experiment** | **2nd Experiment** | **Day** |
| --- | --- | --- | --- |
| Sterilize and Plate Seeds | 5/22/2024 | 6/20/2024 |  |
| Start Germination | 5/25/2024 | 6/23/2024 |  |
| Transfer seedlings, Load EcoFABs | 5/28/2024 | 6/26/2024 | 1 |
| 1st Images collected | 5/31/2024 | 6/29/2024 | 4 |
| Imaging | 6/2/2024 | 7/1/2024 | 6 |
| Imaging | 6/4/2024 | 7/3/2024 | 8 |
| Imaging | 6/6/2024 | 7/5/2024 | 10 |
| Add Copper | 6/7/2024 | 7/6/2024 | 11 |
| Imaging | 6/8/2024 | 7/7/2024 | 12 |
| Imaging | 6/10/2024 | 7/9/2024 | 14 |
| Imaging | 6/12/2024 | 7/11/2024 | 16 |
| Imaging | 6/14/2024 | 7/13/2024 | 18 |
| Imaging | 6/16/2024 | 7/15/2024 | 20 |
| Imaging | 6/18/2024 | 7/17/2024 | 22 |
| Imaging | 6/20/2024 |  | 24 |
| Harvest and Last Imaging | 6/21/2024 | 7/20/2024 | 25 |

**Table S4. Rationale for the removal of plants from the datasets**

| **Experiment** | **Barcode** | **Reason** |
| --- | --- | --- |
| Copper Pilot | FY24EX0009EF015 | Died prematurely |
| 2Part Copper-1st | YY24EX0010EF010 | Root Growth Into Gasket |
| 2Part Copper-1st | YY24EX0010EF013 | Root Growth Outside |
| 2Part Copper-1st | YY24EX0010EF017 | Root Growth Outside |
| 2Part Copper-1st | YY24EX0010EF018 | Image analysis issues |
| 2Part Copper-1st | YY24EX0010EF019 | Imaging Issues |
| 2Part Copper-1st | YY24EX0010EF020 | Root Growth Outside |
| 2Part Copper-1st | YY24EX0010EF024 | Root Growth Outside |
| 2Part Copper-1st | YY24EX0010EF027 | Root Growth Outside |
| 2Part Copper-1st | YY24EX0010EF031 | Root Growth Outside |
| 2Part Copper-1st | YY24EX0010EF038 | Root Growth Outside |
| 2Part Copper-1st | YY24EX0010EF042 | Died prematurely |
| 2Part Copper-2nd | YY24EX0011EF020 | Image analysis issues |
| 2Part Copper-2nd | YY24EX0011EF027 | Image analysis issues |
| 2Part Copper-2nd | YY24EX0011EF034 | Image analysis issues |
| 2Part Copper-2nd | YY24EX0011EF039 | Died prematurely |

**Table S5. Student’s T-Tests comparing Model Test Set RMSE values vs Shuffled (Fake) FRMSE values**

|  | **RMSE_Mean** | **RMSE_Std** | **FRMSE_Mean** | **FRMSE_Std** | **p-value** |
| --- | --- | --- | --- | --- | --- |
| Combined_Root | 4.589151 | 1.343736 | 12.82121 | 3.110612 | 2.83E-19 |
| 1st_Expt_Root | 5.031204 | 1.527078 | 10.68591 | 5.114269 | 2.87E-07 |
| 2nd_Expt_Root | 4.250672 | 2.767813 | 9.370438 | 6.400098 | 0.000169 |
| 1st_Expt_Norm_Root | 0.124017 | 0.038011 | 0.310802 | 0.112023 | 5.07E-12 |
| 2nd_Expt_Norm_Root | 0.090377 | 0.058864 | 0.201689 | 0.147304 | 0.000304 |
| Combined_Norm_Root | 0.110414 | 0.027385 | 0.286641 | 0.065422 | 1.05E-19 |
| 1st_Expt_Scaled_RP | 0.09111 | 0.032268 | 0.156074 | 0.072169 | 3.31E-05 |
| 2nd_Expt_Scaled_RP | 0.079293 | 0.054318 | 0.224964 | 0.141419 | 2.12E-06 |
| Combined_Scaled_RP | 0.091804 | 0.040516 | 0.220277 | 0.072901 | 1.14E-11 |
| 1st_Expt_Shoot | 8.748727 | 2.128058 | 13.24792 | 3.212065 | 3.01E-08 |
| 2nd_Expt_Shoot | 6.552852 | 2.563407 | 13.06759 | 3.723497 | 9.24E-11 |
| Combined_Shoot | 7.667486 | 1.685053 | 13.68406 | 2.454091 | 6.28E-16 |
| 1st_Expt_Norm_Shoot | 0.182211 | 0.045298 | 0.262597 | 0.0806 | 1.32E-05 |
| 2nd_Expt_Norm_Shoot | 0.125203 | 0.048998 | 0.258204 | 0.065055 | 1.64E-12 |
| Combined_Norm_Shoot | 0.179798 | 0.027395 | 0.343392 | 0.057064 | 1.80E-20 |
| 1st_Expt_Scaled_SP | 0.0985 | 0.029926 | 0.208288 | 0.06386 | 8.08E-12 |
| 2nd_Expt_Scaled_SP | 0.069789 | 0.030364 | 0.18499 | 0.06612 | 4.62E-12 |
| Combined_Scaled_SP | 0.083665 | 0.019236 | 0.228849 | 0.043327 | 6.40E-24 |

**Table S6. ANOVA evaluating test sets NRMSE values of models of 1^st^ Experiment, 2^nd^ Experiment and Combined Experiments**

| **Group** |  | **Sum_SQ** | **df** | **F** | **PR(>F)** |
| --- | --- | --- | --- | --- | --- |
| Root | C(Dataset) | 0.269454 | 2 | 6.988469 | 0.001533 |
| Root | Residual | 1.677224 | 87 |  |  |
| Norm_Root | C(Dataset) | 0.240385 | 2 | 6.20738 | 0.00302 |
| Norm_Root | Residual | 1.684568 | 87 |  |  |
| Scaled_RP | C(Dataset) | 1.005826 | 2 | 7.314725 | 0.001158 |
| Scaled_RP | Residual | 5.981556 | 87 |  |  |
| Shoot | C(Dataset) | 0.168414 | 2 | 14.00813 | 5.32E-06 |
| Shoot | Residual | 0.522983 | 87 |  |  |
| Norm_Shoot | C(Dataset) | 0.201869 | 2 | 17.50522 | 4.08E-07 |
| Norm_Shoot | Residual | 0.501639 | 87 |  |  |
| Scaled_SP | C(Dataset) | 0.162729 | 2 | 4.339867 | 0.015974 |
| Scaled_SP | Residual | 1.631085 | 87 |  |  |

**Table S7. Post Hoc Tukey HSD Multiple Comparisons of Models From Table S5 ANOVA Analysis**

| **Models** | **Group 1** | **Group 2** | **MeanDiff** | **p-adj** | **Lower** | **Upper** |
| --- | --- | --- | --- | --- | --- | --- |
| Root | 1st_Expt_Root | 2nd_Expt_Root | 0.0484 | 0.3723 | -0.0371 | 0.1338 |
| Root | 1st_Expt_Root | Combined_Root | -0.0841 | 0.0549 | -0.1696 | 0.0014 |
| Root | 2nd_Expt_Root | Combined_Root | -0.1324 | 0.0011 | -0.2179 | -0.0469 |
| Shoot | 1st_Expt_Shoot | 2nd_Expt_Shoot | -0.0733 | 0.0012 | -0.1211 | -0.0256 |
| Shoot | 1st_Expt_Shoot | Combined_Shoot | -0.1029 | 0 | -0.1506 | -0.0552 |
| Shoot | 2nd_Expt_Shoot | Combined_Shoot | -0.0296 | 0.3066 | -0.0773 | 0.0182 |
| Norm_Root | 1st_Expt_Norm_Root | 2nd_Expt_Norm_Root | 0.0509 | 0.3367 | -0.0348 | 0.1366 |
| Norm_Root | 1st_Expt_Norm_Root | Combined_Norm_Root | -0.0749 | 0.0988 | -0.1606 | 0.0107 |
| Norm_Root | 2nd_Expt_Norm_Root | Combined_Norm_Root | -0.1258 | 0.0021 | -0.2115 | -0.0402 |
| Norm_Shoot | 1st_Expt_Norm_Shoot | 2nd_Expt_Norm_Shoot | -0.0696 | 0.0018 | -0.1163 | -0.0228 |
| Norm_Shoot | 1st_Expt_Norm_Shoot | Combined_Norm_Shoot | -0.1152 | 0 | -0.1619 | -0.0684 |
| Norm_Shoot | 2nd_Expt_Norm_Shoot | Combined_Norm_Shoot | -0.0456 | 0.0575 | -0.0924 | 0.0011 |
| Scaled_RP | 1st_Expt_Scaled_RP | 2nd_Expt_Scaled_RP | -0.1618 | 0.0493 | -0.3232 | -0.0004 |
| Scaled_RP | 1st_Expt_Scaled_RP | Combined_Scaled_RP | -0.256 | 0.0008 | -0.4174 | -0.0946 |
| Scaled_RP | 2nd_Expt_Scaled_RP | Combined_Scaled_RP | -0.0942 | 0.35 | -0.2556 | 0.0673 |
| Scaled_SP | 1st_Expt_Scaled_SP | 2nd_Expt_Scaled_SP | -0.072 | 0.1094 | -0.1563 | 0.0123 |
| Scaled_SP | 1st_Expt_Scaled_SP | Combined_Scaled_SP | -0.1012 | 0.0145 | -0.1855 | -0.0169 |
| Scaled_SP | 2nd_Expt_Scaled_SP | Combined_Scaled_SP | -0.0291 | 0.6891 | -0.1134 | 0.0552 |

**Table S8. System temperature information for two-part copper experiment**

|  |  | **Air** | | **Shoot** | | **Root** | |
| --- | --- | --- | --- | --- | --- | --- | --- |
| **Experiment** | **TimeFrame** | **Mean** | **Std** | **Mean** | **Std** | **Mean** | **Std** |
| 1st Copper | Day | 25.11 | 0.63 | 24.87 | 0.59 | 23.55 | 0.53 |
| 1st Copper | Night | 23.08 | 0.74 | 22.53 | 0.79 | 21.38 | 0.76 |
| 2nd Copper | Day | 25.28 | 0.54 | 25.05 | 0.57 | 23.73 | 0.52 |
| 2nd Copper | Night | 23.34 | 0.79 | 22.82 | 0.85 | 21.65 | 0.8 |

For Tables S8 and S9, the averages of the two readings for each parameter which are recorded every 5 minutes over the course of the experiment were used in these calculations. Raw data can be found in the data addendum.

**Table S9. T-test Results for Temperature Logs for Air, Root and Shoot Readings for 1st and 2nd Experiment**

| **TimeFrame** | **Measurement** | **T-statistic** | **P-value** |
| --- | --- | --- | --- |
| Day | Air | -12.69850361 | 1.40E-36 |
| Night | Air | -12.27835751 | 3.30E-34 |
| Day | Shoot | -13.94871312 | 1.08E-43 |
| Night | Shoot | -12.87753156 | 2.07E-37 |
| Day | Root | -14.79006222 | 7.87E-49 |
| Night | Root | -12.82176083 | 4.18E-37 |

For Tables S8 and S9, the averages of the two readings for each parameter which are recorded every 5 minutes over the course of the experiment were used in these calculations. Raw data can be found in the data addendum.

**Table S10. Student’s T-Tests Comparing Model NRMSE Values of Test Sets Evaluated with Models Using Copper as the Only Input Variable vs. All Recorded Environmental Variables**

|  | **CuOnly_Mean** | **CuOnly_Std** | **All_Mean** | **All_Std** | **p-value** |
| --- | --- | --- | --- | --- | --- |
| Combined_Root | 0.156516 | 0.060405 | 0.156864 | 0.069205 | 0.983527 |
| 1st_Expt_Root | 0.240589 | 0.143036 | 0.250497 | 0.152254 | 0.79595 |
| 2nd_Expt_Root | 0.288949 | 0.18365 | 0.304895 | 0.19712 | 0.746954 |
| 1st_Expt_Norm_Root | 0.237294 | 0.141991 | 0.251641 | 0.152873 | 0.707808 |
| 2nd_Expt_Norm_Root | 0.288198 | 0.183862 | 0.30776 | 0.204818 | 0.69848 |
| Combined_Norm_Root | 0.162367 | 0.0642 | 0.161986 | 0.073372 | 0.982994 |
| 1st_Expt_Scaled_RP | 0.426528 | 0.394743 | 0.474638 | 0.446907 | 0.660185 |
| 2nd_Expt_Scaled_RP | 0.264713 | 0.20715 | 0.276554 | 0.196136 | 0.82095 |
| Combined_Scaled_RP | 0.17054 | 0.086759 | 0.177956 | 0.084245 | 0.73814 |
| 1st_Expt_Shoot | 0.3253 | 0.088419 | 0.327156 | 0.085182 | 0.934297 |
| 2nd_Expt_Shoot | 0.251975 | 0.09181 | 0.255486 | 0.089713 | 0.881464 |
| Combined_Shoot | 0.222393 | 0.042272 | 0.225084 | 0.044291 | 0.810614 |
| 1st_Expt_Norm_Shoot | 0.324721 | 0.088692 | 0.327728 | 0.086407 | 0.894654 |
| 2nd_Expt_Norm_Shoot | 0.255144 | 0.093011 | 0.260455 | 0.087367 | 0.820485 |
| Combined_Norm_Shoot | 0.209541 | 0.02794 | 0.213561 | 0.028191 | 0.581199 |
| 1st_Expt_Scaled_SP | 0.258402 | 0.228337 | 0.246895 | 0.205672 | 0.838215 |
| 2nd_Expt_Scaled_SP | 0.186371 | 0.056689 | 0.196929 | 0.059899 | 0.485964 |
| Combined_Scaled_SP | 0.157233 | 0.02988 | 0.161882 | 0.035217 | 0.583536 |

**Table S11. Pearson correlation with Confidence Intervals and Coefficient of Determination between Shoot Pixel Counts and Fresh weights.**

| **Image Processing*** | **Pearson Correlation** | **Lower CI** | **Upper CI** | **R2** |
| --- | --- | --- | --- | --- |
| Euclidean | 0.85 | 0.77 | 0.91 | 0.73 |
| Euclidean (post smoothing) | 0.85 | 0.77 | 0.91 | 0.73 |
| Sum | 0.85 | 0.77 | 0.9 | 0.72 |
| Top | 0.84 | 0.76 | 0.9 | 0.71 |
| Front | 0.76 | 0.64 | 0.84 | 0.58 |
| Side | 0.7 | 0.56 | 0.8 | 0.49 |

The 71 plants used in the analysis of the 2-part copper experiment. Confidence intervals were determined using Fischer’s z-transformation of the data. Euclidean refers to the square root of the summed squared data from each angle (side, top, front) and sum is simply the three added together. NOTE: While the top angle alone appears to correlate well, it is important to keep in mind that these images were compared on the final day of growth and this may not hold true during earlier stages of growth.

**Appendix** **S1. (separate file)**

Excel Sheet with the following data provided as individual sheets

1. Nutrient Experiment_Data
2. Nutrient_Expt_ANOVA_Tukey_Shoots
3. Nutrient_Expt_ANOVA_Tukey_Roots
4. Copper_Pilot_Experiment_Data
5. TwoPart_Copper_Experiment_Data
6. TwoPart_Copper_Model_Summaries
7. Calculated NRMSE Values
8. Test Groups used to Evaluate 1^st^ Experiment Models
9. Test Groups used to Evaluate 2^nd^ Experiment Models
10. Test Groups used to Evaluate Combined Experiment Models
11. TempLogs_TwoPart_1st
12. TempLogs_TwoPart_2nd
13. PixelCountData
14. Pearson Correlation Information
15. Copper2part_Tukey_TimeSeries
16. UMAP_Statistics
17. Copper2part_RootTSdata
18. Copper2part_ShootTSdata
19. Copper2part_ShootHealthTSdata
20. Copper2part_NDVI_TSdata
